# Substrate discrimination and quality control require each catalytic activity of TRAMP and the nuclear RNA exosome

**DOI:** 10.1073/pnas.2024846118

**Published:** 2021-03-29

**Authors:** Mom Das, Dimitrios Zattas, John C. Zinder, Elizabeth V. Wasmuth, Julien Henri, Christopher D. Lima

**Affiliations:** ^a^Structural Biology Program, Sloan Kettering Institute, Memorial Sloan Kettering Cancer Center, New York, NY 10065;; ^b^Tri-Institutional Training Program in Chemical Biology, Memorial Sloan Kettering Cancer Center, New York, NY 10065;; ^c^HHMI, Memorial Sloan Kettering Cancer Center, New York, NY 10065

**Keywords:** RNA, quality control, RNA decay, exosome, TRAMP

## Abstract

Quality control requires discrimination between functional and aberrant species to selectively target aberrant substrates for destruction. Nuclear RNA quality control in *Saccharomyces cerevisiae* includes the TRAMP complex that marks RNA for decay via polyadenylation followed by helicase-dependent 3′ to 5′ degradation by the RNA exosome. Using reconstitution biochemistry, we show that polyadenylation and helicase activities of TRAMP cooperate with processive and distributive exoribonuclease activities of the nuclear RNA exosome to protect stable RNA from degradation while selectively targeting and degrading less stable RNA. Substrate discrimination is lost when the distributive exoribonuclease activity of Rrp6 is inactivated, leading to degradation of stable and unstable RNA species. These data support a proofreading mechanism in which deadenylation by Rrp6 competes with Mtr4-dependent degradation to protect stable RNA while selectively targeting and degrading unstable RNA.

## Significance

Defects in RNA quality control pathways manifest as disease because they function to selectively remove aberrant or defective species to ensure cellular homeostasis. The RNA exosome and TRAMP complexes encompass key components of the RNA surveillance machinery. Here we show that exoribonuclease activities of Rrp6-associated RNA exosomes protect stable RNA species from TRAMP-mediated polyadenylation and degradation, and that each catalytic activity of the RNA exosome-TRAMP complex contributes to substrate discrimination and degradation of less stable RNA species. Our results highlight a previously unappreciated role for Rrp6 in protecting stable RNA species from degradation and suggest additional mechanistic analogies between RNA and protein quality control pathways with respect to proofreading mechanisms that protect stable species while targeting unstable species for destruction.

Genome-wide transcriptome analyses in eukaryotes have shown that the nuclear RNA exosome acts on a plethora of RNA substrates by degrading aberrant noncoding and coding RNAs and processing the 3′ ends of various RNAs (1–4). The RNA exosome is an essential multisubunit complex that carries out diverse functions in RNA metabolism encompassing normal RNA turnover, processing, maturation, and quality control (5–7). Eukaryotic RNA exosomes include a 9-subunit core (Exo9) that forms a prominent central channel large enough to accommodate single-stranded RNA (8), while exterior surfaces of Exo9 contribute to recruitment of its two 3′ to 5′ exoribonuclease catalytic subunits, the processive exoribonuclease Dis3 (also known as Rrp44) and the distributive exoribonuclease Rrp6, and cofactors. In several structures, Rrp6 binds to the top of the Exo9 core while Dis3 binds to the bottom (9–12). RNA substrates can be engaged by threading RNA partially or completely through the central channel to Rrp6 or Dis3 active sites, respectively (10, 11, 13).

Substrate specificities and activities of the nuclear RNA exosome are modulated by additional factors that associate with the exosome complex (14, 15). Among these conserved eukaryotic factors are Mtr4, an essential 3′ to 5′ DExH RNA helicase, and two nuclear cofactors, Mpp6 and Rrp47, that mediate interactions between the Mtr4 helicase and the nuclear RNA exosome (16–23). Mtr4 is also a core subunit of several complexes that engage RNA substrates prior to degradation. Among these is TRAMP, a complex conserved across eukaryotic evolution; NEXT and PAXT, which appear conserved in metazoans; and MTREC, a complex discovered in fission yeast that includes the Mtr4-like helicase Mtl1 (5, 7).

Budding yeast TRAMP includes one of two nontemplated poly(A) polymerases of the Polβ family, Trf4 or Trf5, and one of two zinc-knuckle RNA-binding proteins, Air1 or Air2 (24–27). TRAMP promotes degradation of RNA transcripts produced by all three RNA polymerases (28) through the addition of short oligo-adenylated tails to the 3′ end of RNAs (24, 25, 29, 30). While TRAMP substrates with longer tails have been observed (24–26), shorter tails are observed in vitro (30, 31) and in vivo (32), presumably because tails get captured by the Mtr4 helicase, thus sequestering the 3′ end from further rounds of adenylation.

Genetic studies in yeast led to the discovery that TRAMP promotes nuclear RNA exosome-dependent decay of a hypomodified initiator methionine tRNA (tRNA_i_^Met^) that lacks the m^1^A58 modification (24–26, 33–36). While this hypomodified initiator methionine tRNA is functional in translation, the absence of 1-methyl on A58 disrupts D- and T-loop interactions to destabilize the tRNA tertiary structure (25; discussed in ref. 37), thereby making it a substrate for TRAMP and the RNA exosome. Additional studies have shown that the TRAMP-exosome surveillance pathway also targets other nuclear RNAs that include mRNAs, snoRNAs, snRNAs, rRNAs, and ncRNAs generated from intergenic spacer regions and cryptic unstable transcripts in yeast (24, 26, 32, 38, 39).

TRAMP presumably targets aberrant or less stable tRNA by polyadenylating the 3′ end to provide a single-stranded RNA extension that is long enough to engage the Mtr4 helicase (30, 31). Once Mtr4 engages the tRNA and RNA exosome, helicase activity likely unfolds the tRNA to translocate its 3′ end through the central channel toward the processive exoribonuclease activities of Dis3, similar to what was observed for other RNA substrates (20–22, 40). However, roles for Rrp6 distributive exoribonuclease activity, the exosome cofactors Mpp6 or Rrp47, or the Exo9 core in degradation of a tRNA substrate remain less clear.

Here we present the biochemical reconstitution of TRAMP-mediated tRNA decay through characterization of nuclear exosome complexes from the budding yeast *Saccharomyces cerevisiae*. Mutations in each catalytic subunit were analyzed individually and in combinations to probe the importance of each activity for substrate discrimination and selective degradation of less stable tRNA species. We show that each catalytic activity of TRAMP and the nuclear RNA exosome are required for substrate discrimination. We find that Mtr4 helicase activity is required when Rrp6 catalytic activities are present; however, exosomes lacking Rrp6 activity lose the ability to discriminate between stable and less stable substrates, leading to accumulation of polyadenylated species that can be degraded by Dis3 independent of Mtr4 helicase activity. These data support a proofreading model for substrate discrimination and nuclear RNA quality control. While TRAMP polyadenylates both stable and less stable RNAs, the poly(A) tails added to stable RNAs are trimmed by the distributive exoribonuclease activities of Rrp6 presumably before the helicase can be engaged. In contrast, less stable RNAs are better substrates for polyadenylation and translocation, presumably bypassing Rrp6 activities by Mtr4-dependent translocation of their 3′ ends to the processive exoribonuclease activities of Dis3.

## Results

### TRAMP Selectively Targets Unmodified tRNA for Degradation by the Nuclear RNA Exosome.

**Fig. 1.**
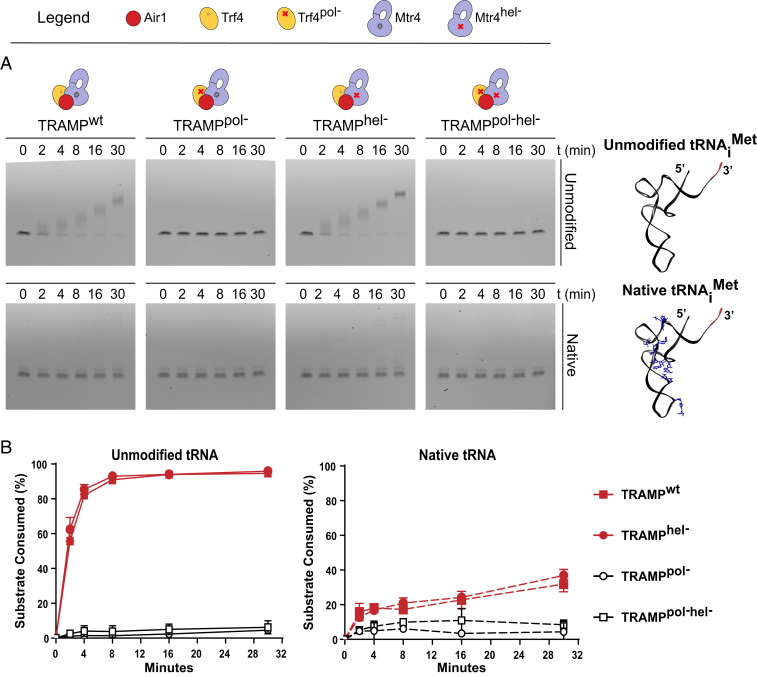
TRAMP selectively polyadenylates unmodified tRNA. (*A*) Time course to detect polyadenylation of unmodified tRNA_i_^Met^ (*Top*) and native deacylated tRNA_i_^Met^ (*Bottom*) by yeast TRAMP and mutants lacking polymerase or helicase activities. RNA was separated by electrophoresis with polyacrylamide TBE-urea gels and detected by SYBR Gold staining. Gel images are representative of three independent experiments. Cartoons above gel panels represent the composition of complexes used, with a red cross indicating an active site mutant. On the right, the ribbon diagram depicts unmodified tRNA_i_^Met^ and yeast native tRNA_i_^Met^ (Protein Data Bank ID code 1YFG), with modified bases shown in blue for native tRNA and 3′-CCA ends depicted in red. Structural representations were generated with Chimera (www.cgl.ucsf.edu/chimera). (*B*) Graphs indicating the percentage of unmodified or native tRNA substrate consumed by TRAMP complexes with time as quantified from experiments in *A*. The mean values of triplicate experiments are plotted, with error bars representing ±1 SD.

Recombinant Air1, Trf4, and Mtr4 were expressed, purified, and reconstituted to form TRAMP. Similar to previous results with hypomodified or unmodified tRNA_i_^Met^ or with unmodified tRNA^Ala^ (24–26, 35), yeast TRAMP polyadenylated unmodified tRNA_i_^Met^, with >80% of the tRNA consumed within 4 min at 30 °C (Fig. 1). In contrast, fourfold-less native deacylated tRNA_i_^Met^ was polyadenylated by TRAMP in the first 4 min. To determine the contributions of TRAMP polymerase and helicase activities to polyadenylation, TRAMP was reconstituted with proteins containing inactivating point mutations in the polymerase and helicase active sites, respectively, to generate TRAMP^pol−^, TRAMP^hel−^, and TRAMP^pol−/hel−^. The Trf4^pol−^ mutant contains two point mutations in its active site, D236N and D238N, that are predicted to inactivate polymerase activity as reported previously (25, 26). The Mtr4^hel−^ mutant contains a E263Q substitution in the DExH motif to inactivate helicase activity by preventing ATP hydrolysis (41, 42). Consistent with prior reports (30), assays with TRAMP^hel−^ revealed slightly elevated levels of polyadenylation compared to wild-type (WT) TRAMP with the unmodified substrate. As expected, TRAMP complexes lacking a functional polymerase (TRAMP^pol−^ or TRAMP^pol−/hel−^) exhibited no detectable polyadenylation activity (Fig. 1 *A* and *B*).

**Fig. 2.**
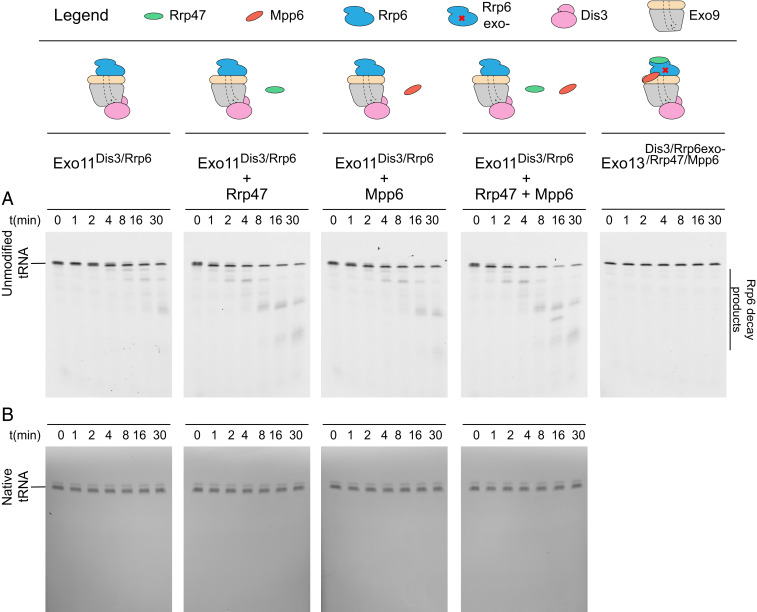
Dis3 does not degrade tRNA in the nuclear exosome. (*A*) Time course to detect degradation of 5′-fluor-tRNA_i_^Met^ by reconstituted nuclear exosomes (Exo11^Dis3/Rrp6^) in the absence or presence of Rrp47 and/or Mpp6, or in the presence of an exosome lacking Rrp6 catalytic activity (rightmost panel). RNA was detected by fluorescein fluorescence. Cartoons for RNA exosome components used in reactions are displayed above the gel images. (*B*) Time course to detect degradation of native tRNA_i_^Met^ by reconstituted nuclear exosomes (Exo11^Dis3/Rrp6^) in the absence or presence of Rrp47 and/or Mpp6. RNA was detected by fluorescence after staining with SYBR Gold. The gel images in *A* and *B* are representative of three independent experiments.

We next tested whether nuclear exosomes containing Rrp6 and Dis3 (Exo11^Dis3/Rrp6^) could degrade unmodified or native tRNA. Neither substrate was efficiently degraded under the conditions tested, indicating that both species are folded and stable enough to protect their 3′ ends from the exoribonuclease activities of Rrp6 and Dis3 (Fig. 2). Consistent with earlier studies (20, 21), the addition of protein cofactors Mpp6 and/or Rrp47 stimulates Rrp6 activity, resulting in enhanced degradation of unmodified tRNA with decay products consistent with Rrp6 distributive trimming activity (Fig. 2*A*). Rrp6-dependent decay products were no longer observed with exosome complexes containing catalytically inactive Rrp6 (Exo13^Dis3/Rrp6exo−/Rrp47/Mpp6^; Fig. 2*A*). Importantly, the native tRNA substrate remained stable in the presence of each nuclear exosome complex (Fig. 2*B*).

**Fig. 3.**
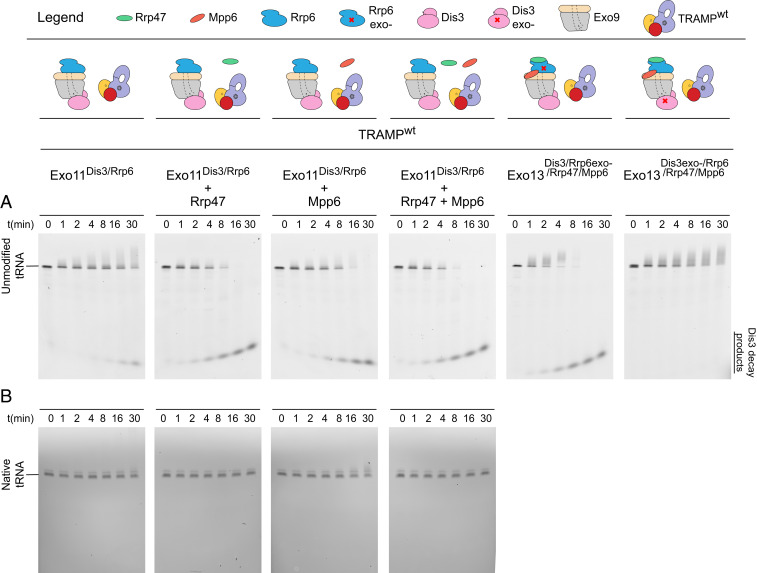
Dis3-dependent decay of unmodified tRNA by TRAMP and the nuclear exosome. (*A*) Time course to detect degradation of unmodified 5′-fluor-tRNA_i_^Met^ by Exo11^Dis3/Rrp6^ in the presence of TRAMP and/or Rrp47 and/or Mpp6, or in the presence of exosomes lacking Dis3 or Rrp6 catalytic activity (two rightmost panels). RNA was detected by fluorescein fluorescence. Cartoons for the exosome and TRAMP components used in the reactions are displayed above the gel images. (*B*) Time course to detect degradation of native tRNA_i_^Met^ by TRAMP and the nuclear exosome. RNA was detected by fluorescence after staining with SYBR Gold. Gel images in *A* and *B* are representative of three independent experiments.

We next combined TRAMP with nuclear RNA exosomes containing the Exo9 core, Rrp6, and Dis3 (Fig. 3). For unmodified tRNA, substrate polyadenylation and 4- to 5-nt decay products of Dis3 activity were detected (Fig. 3*A*); however, polyadenylation or degradation of the native tRNA was not detected (Fig. 3*B*). Furthermore, the addition of Mpp6 and Rrp47, cofactors that similarly stimulate Mtr4 helicase activity and bridge it to the exosome (19–21), increased Dis3 products for unmodified tRNA (Fig. 3*A*), but not for native tRNA (Fig. 3*B*). Dis3-dependent degradation of unmodified tRNA was independent of Rrp6 exonuclease activity, and 4- to 5-nt decay products were not generated with exosome complexes lacking Dis3 exoribonuclease activity (Exo13^Dis3exo−/Rrp6/Rrp47/Mpp6^; Fig. 3*A*).

The results presented thus far suggest that TRAMP selectively targets the less stable unmodified tRNA for polyadenylation and degradation by nuclear RNA exosomes when Rrp6, Dis3, and at least one of the two nuclear cofactors Mpp6 and Rrp47 are present. The observation that TRAMP-mediated RNA degradation is enhanced by Mpp6 or Rrp47 is consistent with recent evidence demonstrating that each cofactor contributes to the recruitment of Mtr4 to the RNA exosome, which is required for helicase-dependent RNA decay of model double-stranded RNA substrates with single-stranded 3′ poly(A) tails (20, 21). It is noteworthy that stimulation of Rrp6 activity by Rrp47 and Mpp6 resulted in accumulation of Rrp6-dependent products with little to no 4- to 5-nt decay products of Dis3 exoribonuclease activity observed (Fig. 2*A*), suggesting that TRAMP polyadenylation may bypass or counter Rrp6 processing activities to promote Dis3-dependent RNA decay in the context of a fully assembled nuclear RNA exosome-TRAMP complex (Fig. 3*A*). Consistent with this, the smear of polyadenylated substrate is fully degraded in exosomes lacking Rrp6 activity, as evidenced by accumulation of Dis3-dependent 4- to 5-nt products, while the smear of polyadenylated substrate remains in exosomes lacking Dis3 activity (Fig. 3*A*).

### TRAMP Activities Are Required for Degradation of Unmodified tRNA.

**Fig. 4.**
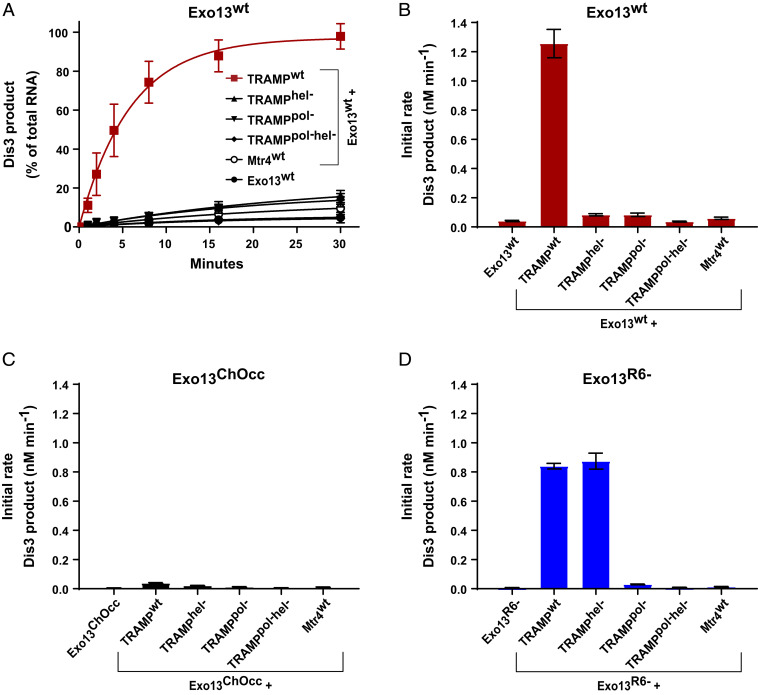
Catalytic requirements for TRAMP-exosome–dependent tRNA decay. (*A*) Graph depicting the time course of Dis3 product accumulation as a fraction of total RNA for unmodified 5′-fluor-tRNA_i_^Met^ in the presence of Exo13^Dis3/Rrp6/Rrp47/Mpp6^ (Exo13^wt^) and in the absence or presence of TRAMP, TRAMP complexes reconstituted with helicase or polymerase mutations, and Mtr4. Assays were performed in triplicate, and mean values are shown. Error bars represent ±1 SD. Representative gel images are shown in *SI Appendix*, Fig. S1. (*B*) Initial rates of Dis3 product formation derived within the linear range from the data in *A*. Mean values are shown, and error bars represent ±1 SD. (*C*) Initial rates of Dis3 product formation derived within the linear range from assays to detect degradation of 5′-fluor-tRNA_i_^Met^ with exosome complexes containing channel occlusions, Exo13^Channel^ ^Occlusion/Dis3/Rrp6/Rrp47/Mpp6^ complex (Exo13^ChOcc^), in the absence or presence of TRAMP, TRAMP complexes reconstituted with helicase or polymerase mutations, or Mtr4. Assays were performed in triplicate and mean values are shown, with error bars representing ±1 SD. Representative gel images are shown in *SI Appendix*, Fig. S3. (*D*) Initial rates of Dis3 product formation derived within the linear range from assays to detect degradation of 5′-fluor-tRNA_i_^Met^ with exosome complexes containing the Rrp6 catalytic mutant, Exo13^Dis3/Rrp6exo-/Rrp47/Mpp6^ (Exo13^R6-^), in the absence or presence of TRAMP, TRAMP complexes reconstituted with helicase or polymerase mutations, or Mtr4. Assays were performed in triplicate, and mean values are shown, with error bars representing ±1 SD. Representative gel images are shown in *SI Appendix*, Fig. S4*A*.

To determine whether TRAMP polymerase and helicase activities contribute to degradation of unmodified tRNA in the context of nuclear exosomes with Mpp6 and Rrp47 (Exo13^Dis3/Rrp6/Rrp47/Mpp6^), the activities of nuclear exosomes were assessed in the absence and presence of TRAMP, TRAMP^pol−^, TRAMP^hel−^, TRAMP^pol−/hel−^, Trf4/Air1, and Mtr4 (Fig. 4*A* and *SI Appendix*, Fig. S1). Products of Rrp6 were again evident when unmodified tRNA was incubated with nuclear exosomes in the absence of TRAMP. In contrast, combining TRAMP and the nuclear exosome resulted in degradation of RNA by Dis3, as evidenced by diminished Rrp6 products and the appearance of Dis3 products (Fig. 4*A* and *SI Appendix*, Fig. S1). The addition of TRAMP^pol−^ or TRAMP^hel−^ resulted in the appearance of Rrp6 products and detectable Dis3 products, while the addition of TRAMP^pol−/hel−^ or Mtr4 resulted in degradation patterns comparable to those seen in samples lacking TRAMP (*SI Appendix*, Fig. S1).

These effects were quantified by integrating the products of Dis3 activity to obtain initial rates (Fig. 4). Compared to Exo13^Dis3/Rrp6/Rrp47/Mpp6^ or reactions also containing inactivated TRAMP, the addition of WT TRAMP to nuclear exosomes increased Dis3 activity by at least 30-fold (Fig. 4*B*). The polymerase core of TRAMP (Trf/Air) can polyadenylate RNA substrates in vitro (25, 43). However, the addition of the Trf4/Air1 heterodimer to nuclear exosomes resulted in only a fourfold increase in Dis3 products (*SI Appendix*, Fig. S1), presumably because interactions between the RNA exosome and TRAMP are dependent on the Mtr4 protein. These data show that maximal degradation of unmodified tRNA is achieved when the polymerase and helicase activities of TRAMP are present and only in complexes that include one or more cofactors that bridge Mtr4 to the RNA exosome.

### Dis3 and the RNA Exosome Central Channel Are Required for Degradation of Unmodified tRNA.

Structural data revealed two conformations of the Dis3 enzyme in the context of the *S. cerevisiae* exosome: one in which short 3′ RNA tails bypass the central channel to directly access the Dis3 exoribonuclease active site and one in which longer 3′ tails span the through-channel route to reach the Dis3 exonuclease active site (9, 10, 12, 44–46). Despite evidence suggesting that the central channel of the noncatalytic exosome core is important for Dis3-dependent degradation in vitro and in yeast (13, 47), a direct access route to Dis3 may contribute to decay, as suggested by several studies (45, 48, 49). To first assess the importance of Dis3, Exo13^Dis3exo−/Rrp6/Rrp47/Mpp6^ was reconstituted with Dis3 D551N, a mutation that inactivates Dis3 exonuclease activity (50). Exo13^Dis3exo−/Rrp6/Rrp47/Mpp6^ was unable to degrade unmodified tRNA in the presence of TRAMP and TRAMP variants, although some Rrp6 decay products were evident when TRAMP polyadenylation activity was absent (*SI Appendix*, Fig. S2). To determine whether the exosome central channel is important, exosomes were reconstituted with a channel occlusion made with an insertion of 11 (ELGESEGESEG) amino acid residues in Rrp45, a subunit of the Exo9 PH-like ring, as characterized previously (13). Occlusion of the central channel inhibited Dis3-dependent degradation of the unmodified tRNA even in the presence of WT TRAMP (Fig. 4*C* and *SI Appendix*, Fig. S3). These observations are consistent with Mtr4-dependent translocation of the substrate through the exosome central channel. It is noteworthy that Exo13^Dis3exo−/Rrp6/Rrp47/Mpp6^ exosomes are similar to channel-occluded exosomes (Exo13^Channel^ ^Occlusion/Dis3/Rrp6/Rrp47/Mpp6^) with respect to generating fewer Rrp6-dependent decay products (cf. *SI Appendix*, Figs. S2 and S3). This result is consistent with previous findings that RNA molecules that are long enough to traverse the central channel and bind in the Dis3^exo−^ active site, occlude the channel, and inhibit Rrp6 (11–13).

### Contributions of Rrp6 and Mtr4 Helicase Activities to Decay.

We next characterized mutations within Rrp6 and the Mtr4 helicase that disrupt exoribonuclease and ATPase activities, respectively. Exonuclease-inactive Rrp6 D238N (Rrp6^exo−^) was reconstituted to form Exo13^Dis3/Rrp6exo−/Rrp47/Mpp6^ (21). This complex generated no detectable products of Rrp6 activity and was unable to degrade unmodified tRNA in the absence of TRAMP (cf. *SI Appendix*, Figs. S1 and S4*A*). Combining TRAMP with Exo13^Dis3/Rrp6exo−/Rrp47/Mpp6^ resulted in RNA species larger than the tRNA substrate prior to and coincident with the accumulation of Dis3 products (*SI Appendix*, Fig. S4*A*). While larger and presumably polyadenylated intermediates were observed, unmodified tRNA was degraded, with initial rates comparable to those of Exo13 WT (*SI Appendix*, Fig. S4*B*). These data suggest that Rrp6 activities are not required to degrade unmodified tRNA.

Next, TRAMP^pol−^ and TRAMP^hel−^ were combined with Exo13^Dis3/Rrp6exo−/Rrp47/Mpp6^ (*SI Appendix*, Fig. S4*A*). The addition of TRAMP^pol−^ diminished the rate of decay, but unmodified tRNA was readily degraded in reactions containing TRAMP^hel−^ with rates of Dis3 product accumulation comparable to rates observed for TRAMP in the presence of Exo13 WT (Fig. 4*B*) or Exo13^Dis3/Rrp6exo−/Rrp47/Mpp6^ (Fig. 4*D*). These results suggest that Mtr4 helicase activity is dispensable in the absence of Rrp6 exonuclease activity, and that helicase activity is only required to degrade this tRNA substrate when Rrp6 exoribonuclease activity is intact.

Our results highlight an interplay between the catalytic activities of TRAMP, Dis3, and Rrp6 for tRNA substrate decay in vitro. In addition, our data also illustrate that tRNA degradation by the RNA exosome can occur in the absence of helicase activity when Rrp6 exoribonuclease activity is absent.

We next assessed whether these requirements for substrate degradation by the TRAMP-nuclear exosome could be extended to other RNA species. To this end, total RNA was extracted from yeast strains containing either WT or exoribonuclease-inactive Rrp6 (Rrp6^exo−^) or lacking Rrp6 completely (*rrp6Δ*). Extracted RNA was then ribo-depleted and subjected to RNA-sequencing analysis. The sequencing data identified coding and noncoding RNA transcripts that were differentially expressed in the three strains. Rrp6 and its 3′ to 5′ exonuclease activity were shown to contribute to quality control and processing of various types of nuclear RNAs (6, 51–53), and our sequencing analysis further confirms that Rrp6 contributes to this process.

**Fig. 5.**
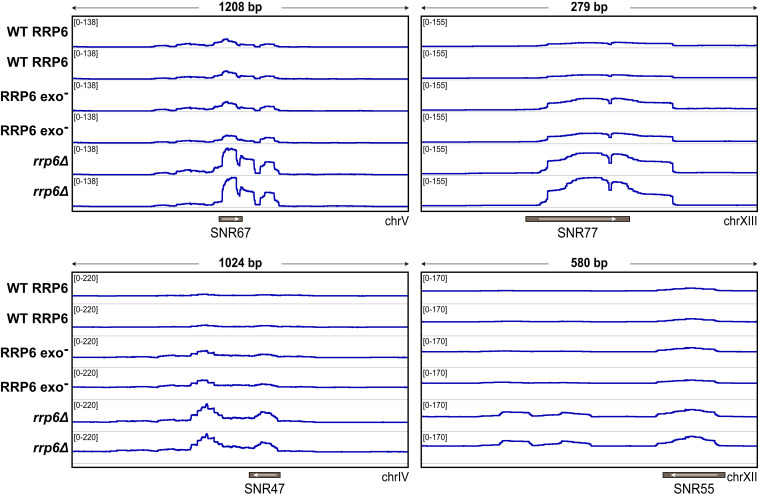
Comparison of RNA abundance between Rrp6 and Rrp6 variants. Integrative Genomics Viewer browser tracks of snoRNA genes *SNR67*, *SNR77*, *SNR47*, and *SNR55* of RNA sequencing data derived from yeast strains with WT Rrp6, exoribonuclease-inactive (Rrp6^exo−^), or Rrp6 knockout (*rrp6Δ*), showing that transcript abundance is lower in WT and Rrp6^exo−^ but not in *rrp6Δ*. Data are derived from two independent biological replicates. The *y*-axis represents normalized reads in reads per kilobase of transcript per million (RPKM).

Similar to in vitro data with tRNA, four representative C/D box snoRNAs—*SNR67*, *SNR77*, *SNR47*, and *SNR55*—were either less abundant or not observed in strains with WT or exonuclease-inactive Rrp6 (Fig. 5) but were more abundant in the *rrp6Δ* strain, suggesting that their levels are dependent on Rrp6 protein but not its exoribonuclease activity. While localization of exosomes might differ in the absence of Rrp6 protein, a recent study showed that depletion of Rrp6 did not alter nuclear transport or subcellular localization of Dis3 or other core exosome subunits (54). These results are also consistent with prior studies in which a catalytically inactive Rrp6 reduced the accumulation of poly(A)^+^ RNAs when expressed in *rrp6Δ* yeast strains (55). In contrast, some RNAs (represented by *SNR13* and *SNR73* in *SI Appendix*, Fig. S5*A*) were observed at low abundance in WT strains but not in strains with exoribonuclease-inactive Rrp6 or *rrp6Δ*, suggesting that these RNAs are dependent on the Rrp6 protein and its catalytic activity. Consistent with our observations, *SNR13* was previously reported to accumulate in cells lacking Rrp6 (39). Other RNAs, as represented by *SNR69* and *COS4* (*SI Appendix*, Fig. S5*B*), remain at similar levels in all three strains, although 3′ extensions are apparent for *SNR69* in strains lacking Rrp6 protein or its catalytic activity, indicating that some substrates may be dependent on Rrp6 for processing but not for degradation.

### Rrp6 Exoribonuclease Activity Protects Native tRNA from Decay.

Since Mtr4 helicase and Rrp6 exoribonuclease activities are dispensable for Dis3-dependent decay of unmodified tRNA, we next assessed whether these activities play a role in discriminating between unmodified and deacylated fully modified native tRNA_i_^Met^ in assays that analyzed substrate stability by measuring the exponential decay of substrate. For native tRNA_i_^Met^ decay, nonlinear regression analysis was performed (*Methods*) using a double-exponential model of decay (*SI Appendix*, Figs. S6 *B* and *D* and S7 and Table S1). However, for degradation of unmodified tRNA_i_^Met^, a single exponential decay equation was sufficient to fit data with one exception, as noted below (*SI Appendix*, Figs. S6 *C* and *E* and S7 and Table S1). Unless noted otherwise, we discuss the dominant rate obtained from data analyzed with a two-phase equation if that rate accounts for >75% of the substrate consumed. We do not report numerical values of substrate half-life in reactions in which the substrate remained stable throughout the time course of our assay.

**Fig. 6.**
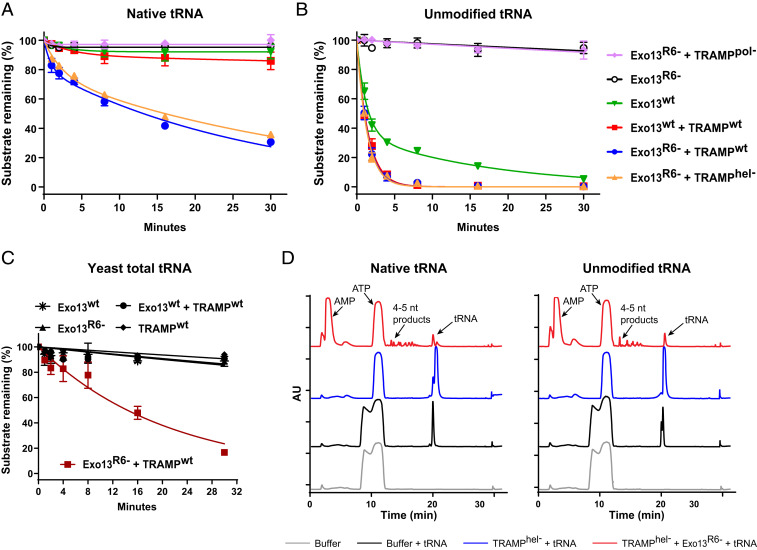
Catalytically inactive Rrp6 activates the exosome for tRNA decay. The graphs depict substrate remaining from a time course for degradation of native deacylated tRNA_i_^Met^ (*A*) or unmodified 5′-fluor-tRNA_i_^Met^ (*B*) by the indicated nuclear exosome and TRAMP complexes with or without mutations that disrupt Rrp6 exoribonuclease or Mtr4 helicase activities. Assays were performed in triplicate, and mean values shown, with error bars representing ±1 SD. Representative gel images for native tRNA_i_^Met^ are shown in *SI Appendix*, Fig. S6*A*. Gel images for degradation of unmodified 5′-fluor-tRNA_i_^Met^ with Exo13^wt^ or Exo13^R6−^ are shown in *SI Appendix*, Figs. S1 and S4*A*, respectively. (*C*) Graphs depicting substrate remaining for degradation of tRNA extracted from yeast by indicated exosome and TRAMP complexes with or without mutations that disrupt Rrp6 exoribonuclease activity. Assays were performed in triplicate, and mean values are shown, with error bars representing ±1 SD. Data were fit to a one-phase decay model (*Methods*). Representative gel images are shown in *SI Appendix*, Fig. S8*B*. (*D*) HPLC analysis of Dis3-dependent degradation products. Reactions contained either deacylated native tRNA_i_^Met^ or unmodified tRNA_i_^Met^ with buffer (black trace), TRAMP^hel−^ (blue trace), or TRAMP^hel−^ with Exo13^Dis3/Rrp6exo-/Rrp47/Mpp6^ (Exo13^R6−^) (red trace). Absorbance was monitored at 260 nm for detection of nucleotides that included 4- to 5-nt RNA decay products. A buffer-only trace at 260 nm is shown as a reference (gray).

Native tRNA_i_^Met^ remained stable when incubated with Exo13^Dis3/Rrp6/Rrp47/Mpp6^ (Fig. 6*A* and *SI Appendix*, Fig. S6*A*). Conversely, unmodified tRNA was degraded in two phases via an Rrp6-mediated process, as no Dis3 products were detected (Fig. 6*B* and *SI Appendix*, Fig. S1). The faster phase had a half-life of 0.86 min and accounted for 64% of the substrate, and the remainder was degraded with a half-life of 12 min (*SI Appendix*, Fig. S6 *C* and *E*). Both native and unmodified tRNA remained stable in the presence of Exo13^Dis3/Rrp6exo−/Rrp47/Mpp6^, which lacked Rrp6 catalytic activity (Fig. 6 *A* and *B* and *SI Appendix*, Fig. S6 *D* and *C*).

We next measured the stability of native tRNA in the presence of TRAMP and Exo13^Dis3/Rrp6/Rrp47/Mpp6^. While native tRNA remained stable (Fig. 6*A* and *SI Appendix*, Fig. S6 *A* and *D*), unmodified tRNA was degraded, with a measured half-life of 1.0 min (Fig. 6*B* and *SI Appendix*, Fig. S6*C*). Disappearance of the unmodified tRNA coincided with accumulation of Dis3 products, suggesting that degradation was dependent on Dis3 exoribonuclease activity (*SI Appendix*, Fig. S1). Consistent with prior results using unmodified tRNA, native tRNA was destabilized and readily degraded (half-life of 19 min; *SI Appendix*, Fig. S6*D*) when incubated with TRAMP and exosomes that lacked Rrp6 exoribonuclease activity (Fig. 6 *A* and *B* and *SI Appendix*, Figs. S6*A* and S4*A*). Furthermore, helicase activity was dispensable for degradation of native tRNA when Rrp6 activity was absent (half-life of 26 min; *SI Appendix*, Fig. S6*D*). Degradation of unmodified tRNA by exosomes deficient in Rrp6 activity was also independent of helicase activity, with RNA half-lives of <1 min observed in the presence or absence of helicase activity (Fig. 6*B* and *SI Appendix*, Fig. S6*C*). In contrast, TRAMP polymerase activity was required to degrade either substrate by Exo13^Dis3/Rrp6exo−/Rrp47/Mpp6^ (Fig. 6 *A* and *B* and *SI Appendix*, Figs. S4*A* and S6*A*). To confirm that degradation of native tRNA was catalyzed by Dis3, ion-pair reverse-phase high-performance liquid chromatography (HPLC) (Fig. 6*D*) was used to detect decay products characteristic of Dis3 catalytic activity for both native and unmodified tRNA, as described previously (12).

Data thus far suggest a model wherein TRAMP-mediated decay of native tRNA is inhibited by Rrp6 exoribonuclease activity. To extend these results, RNA was isolated from budding yeast and further purified to yield total tRNA and other small ribosomal RNA species, like mature 5.8S and 5S (*SI Appendix*, Fig. S8*A*). These RNAs were incubated in the presence of recombinant TRAMP and the nuclear exosome (Exo13^Dis3/Rrp6/Rrp47/Mpp6^) or TRAMP and exosomes that lacked Rrp6 activity (Exo13^Dis3/Rrp6exo−/Rrp47/Mpp6^) (*SI Appendix*, Fig. S8*B*). TRAMP and the nuclear exosome participate in the surveillance, processing, and modification of a variety of rRNAs (26, 32, 34, 56–59), including maturation of the large ribosomal subunit 5.8S rRNA by 3′ end processing of its larger precursor 5.8S+30 (60–63). Extracted mature 5.8S rRNA (∼160 nt) includes an unstructured 3′ end of 21 nt (64) that was presumably trimmed by Rrp6 to an intermediate 5.8S(i) (∼140 nt) in the presence of WT Exo13^Dis3/Rrp6/Rrp47/Mpp6^, because this activity was not observed in exosomes lacking Rrp6 ribonuclease activity. In the presence of TRAMP, however, both 5S (∼120 nt) and 5.8S rRNAs were targeted for polyadenylation and Dis3-dependent degradation by the nuclear RNA exosome, as loss of these RNAs was independent of Rrp6 activity (*SI Appendix*, Fig. S8*B*). When the stability of the total tRNA pool was assessed, similar to results observed using native tRNA_i_^Met^, the total tRNA pool remained stable in the presence of WT TRAMP and the nuclear exosome but was destabilized and degraded in the absence of Rrp6 exoribonuclease activity (half-life of 14.3 ± 1.1 min; Fig. 6*C*).

## Discussion

**Fig. 7.**
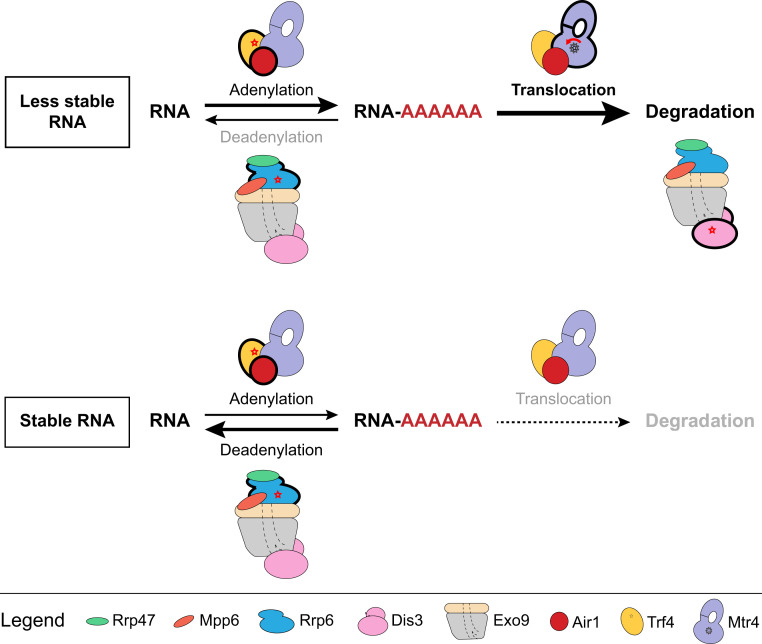
Model for selectivity and specificity in the degradation of unstable or stable RNA. RNA adenylation outpaces Rrp6 deadenylation to enable capture, translocation, and degradation of less stable RNA. In contrast, Rrp6 deadenylation activities protect stable RNA species from helicase-dependent degradation by the RNA exosome.

Quality control pathways discriminate between molecules to selectively target aberrant substrates for degradation. Previous studies have illuminated the importance of TRAMP and the RNA exosome in nuclear RNA quality control (24–26, 29, 32–34, 36, 39, 65–68). Our study supports a model for substrate discrimination and RNA quality control that relies on each of the catalytic activities of TRAMP and the nuclear exosome to selectively degrade less stable substrates while protecting more stable species from degradation (Fig. 7).

Our in vitro data suggest that polyadenylation is required for TRAMP-mediated Dis3-dependent decay of unmodified tRNA by the nuclear RNA exosome. This result is similar to that reported by prior studies that identified in vivo requirements for degradation of hypomethylated tRNA_i_^Met^ (25, 34), but it differs somewhat from work indicating that Trf4 polymerase activity is dispensable for exosome-dependent decay (24, 29). Importantly, TRAMP-mediated decay in the context of WT nuclear exosomes also depends on TRAMP helicase activity, a result consistent with work showing that polyadenylated RNA species accumulate in cells depleted of Mtr4 (69). We found that TRAMP-mediated RNA decay was reduced in the absence of exosome cofactors Mpp6 and Rrp47, in agreement with findings that the Mtr4 helicase is recruited to the nuclear exosome via interactions with Rrp47 and/or Mpp6 (18–23).

The experiments presented here suggest that each catalytic activity of TRAMP and the nuclear RNA exosome is required to selectively degrade unmodified tRNA while leaving native tRNA intact (Fig. 7). These activities include polyadenylation by Trf4, translocation by Mtr4, deadenylation by Rrp6, and degradation by Dis3. Consistent with this, a nuclear exosome lacking Dis3 and Rrp6 exoribonuclease activities failed to degrade unmodified tRNA (*SI Appendix*, Fig. S9). While Rrp6 inactivation had little impact on the overall rate of decay for unmodified RNA, inactivation of Rrp6 destabilized native tRNA. This suggests that Rrp6 deadenylation activities may protect native tRNAs and other RNAs from TRAMP-mediated decay. Along these lines, Rrp6 was recently shown to discriminate against CCA 3′ termini, thereby preventing unwanted degradation of the native tRNA pool (70). It is noteworthy that similar mechanisms appear operative in *Escherichia coli*, as RNase II was reported to rapidly degrade 3′-poly(A) tails of some RNAs, thereby shielding them from digestion by other exonucleases (71).

Our in vitro data reveal that Mtr4 translocation and/or helicase activity competes with Rrp6-mediated trimming to protect stable RNA species while targeting less stable RNAs for degradation. Consistent with this, substrate discrimination between stable and less stable RNA species is reduced on Rrp6 inactivation in a process that no longer requires Mtr4 helicase activity. As native tRNA becomes unstable in the presence of exosomes without Rrp6 ribonuclease activity, it is tempting to speculate a kinetic proofreading function for Rrp6 wherein natively folded RNAs are protected from spurious polyadenylation by Rrp6-mediated deadenylation before these RNAs can be captured by Mtr4 (Fig. 7). A similar kinetic proofreading model has been proposed for the 26S proteasome to promote selective degradation of substrates (72).

The most likely path for RNA involves the central channel, as occlusion of the channel inhibits Rrp6-mediated trimming and formation of Dis3 products. While alternative channel-independent RNA processing pathways are supported by recent in vivo studies (4, 45, 49), our observations are consistent with prior studies indicating the importance of the central channel for RNA decay (9, 12, 13) and with recent studies showing that Mtr4 docks on top of the exosome core to guide RNA into the central channel (18, 20–22).

Analogies between the proteasome and exosome have been noted (73, 74), and our data suggest that they could be extended. In protein quality control, protein substrates are targeted, posttranslationally modified with ubiquitin, and shuttled to the proteasome, where they are interrogated and released by the ubiquitin deconjugation activities of Usp14 (75) or captured and unfolded by the AAA^+^ ATPase ring of the 19S regulatory particle for delivery into the 20S proteolysis chamber, where they are degraded (76, 77). For TRAMP-nuclear exosome–mediated RNA quality control, RNA substrates are targeted, posttranscriptionally modified by the poly(A) polymerase, and shuttled to the exosome, where they can be interrogated and released by the deadenylation activities of Rrp6 or captured and translocated by the Mtr4 helicase for delivery into the exosome for degradation by Dis3.

It is noteworthy that the nuclear exosome can be activated in vitro by mutation of Rrp6, because inactivation of the Usp14 deubiquitinase leads to activation of the proteasome (75). We posit that Rrp6 inhibition could provide the TRAMP-nuclear exosome complex with additional opportunities to engage substrates for translocation by Mtr4 and degradation via Dis3, perhaps relevant to pathologies resulting from inactivating mutations in subunits of the RNA exosome (78).

## Methods

### Protein Purification and Complex Reconstitution.

Cloning, expression, purification of exosome subunits, and reconstitution of various complexes have been described previously (11–13, 20). *S. cerevisiae* Mpp6, Rrp47, and Mtr4 (WT and E263Q catalytic mutant, prepared by site-directed mutagenesis) were cloned into pRSF-Duet1 with an N-terminal Smt3-fusion tag and then transformed into *E. coli* BL21 (DE3) RIL (Novagen). Recombinant protein expression and purification were performed as described previously (20). The Air1 protein component of the yeast-TRAMP complex was truncated at the C terminus by 40 amino acids for expression while preserving the major determinants required for interaction with Trf4 and RNA binding (43). Air1/Trf4, both WT and catalytically inactive double mutant (Trf4 D236N D238N), were cloned into pET-Duet1 vector with an N-terminal hexahistidine tag on Air1 and transformed into *E. coli* BL21 (DE3) (Novagen). Recombinant expression of the Air1/Trf4 heterodimer was induced with 0.5 mM isopropyl-β-d-thiogalactoside and 100 µM zinc chloride with overnight shaking at 20 °C. Cells were harvested by centrifugation and suspended in lysis buffer (20 mM Tris⋅Cl pH 8.0, 500 mM NaCl, 10 mM imidazole, 3 mM beta-mercaptoethanol (BME), 0.1% vol/vol IGEPAL, 10 µg/mL lysozyme, 1 mM phenylmethylsulfonyl fluoride, 10 µg/mL DNase I, and 5 mM MgCl_2_). Cells were lysed by sonication and fractionated by centrifugation at 40,000 × *g* at 4 °C for 45 min to remove insoluble matter. The supernatant was applied to Ni-NTA resin (Qiagen) and washed with 10 column volumes of wash buffer (20 mM Tris⋅Cl pH 8.0, 350 mM NaCl, 3 mM BME and 10 mM imidazole). Protein was eluted with three column volumes of wash buffer that also contained 200 mM imidazole and applied to a Superdex 200 gel filtration column (GE Healthcare) equilibrated in buffer containing 20 mM Tris⋅Cl pH 8.0, 350 mM NaCl, and 3 mM BME. Fractions containing Air/Trf were identified by sodium dodecyl sulfate polyacrylamide gel electrophoresis (SDS-PAGE), pooled, and exchanged into buffer comprising 20 mM Tris-Cl pH 8.0, 200 mM NaCl, and 3 mM BME before being applied to a MonoQ column (GE Healthcare). Proteins were eluted with a gradient of 200 mM to 1,000 mM NaCl, fractions were analyzed by SDS-PAGE, pooled, concentrated to 10 mg/mL by centrifugation using Amicon YM-30 filtration units (EMD Millipore), flash-frozen in liquid N_2_, and stored at −80 °C. To reconstitute TRAMP complexes, a 1.2-fold molar excess of purified Mtr4 was mixed with purified His_6X_-Air1/Trf4 in buffer containing 200 mM NaCl, 20 mM Tris pH 8.0, 1 mM MgCl_2_, and 1 mM dithiothreitol (DTT) for 30 min on ice; dialyzed with a low-salt buffer (100 mM NaCl) for 3 to 4 h at 4 °C; and then applied to a Superdex 200 column in the same buffer. Fractions containing TRAMP were identified by SDS-PAGE, and relevant fractions were pooled, concentrated to 5 to 10 mg/mL, and stored at −80 °C until further use.

### RNA Synthesis and Purification.

Unmodified *S. cerevisiae* tRNA_i_^Met^ includes a guanosine at position 1 instead of adenosine at the 5′ end for efficient in vitro transcription by T7 RNA polymerase. tRNA in pHG300 vector (a gift from Eckhard Jankowsky) was linearized with BstNI, purified by phenol-chloroform extraction, and in vitro transcribed as run-off transcription with T7 RNA polymerase (produced in-house). Transcripts were separated on a 10% polyacrylamide/8 M urea gel and visualized by UV shadowing. The RNA band was excised from the gel, crushed, and eluted in buffer containing 500 mM ammonium acetate and 1 mM EDTA at 37 °C overnight. The RNA product was further extracted with 1-butanol, precipitated with ethanol, resuspended in nuclease-free water, and stored at −80 °C until further use. The tRNA was dephosphorylated with calf intestinal alkaline phosphatase (New England BioLabs), phenol-extracted, ethanol-precipitated, dissolved in nuclease-free water, and labeled with fluorescein as described previously (79). Before use, tRNA was refolded in 10 mM Tris⋅Cl pH 7.5 by heating to 80 °C for 2 min, followed by incubation at 60 °C for 2 min, addition of MgCl_2_ to a final concentration of 10 mM, and subsequent cooling at room temperature for 10 min. It was then stored on ice until the start of the assay. Yeast deacylated native tRNA_i_^Met^ was purchased as a lyophilized powder from tRNA Probes (catalog no. MI-03) and resuspended in nuclease-free water, and the stock was stored at −80 °C until further use.

Total tRNA was isolated from haploid *S. cerevisiae* strain containing individual *rrp6Δ::KanMX* gene deletion obtained from the *Saccharomyces* Gene Deletion Project collection (Open Biosystems) and was in the BY4741/BY4742 background (S288C, *his3Δ1 leu2Δ0 ura3Δ0 met15Δ0*). This haploid *rrp6Δ* strain was transformed with a pRS413 *RRP6* vector containing RRP6 under control and behind its native promoter and plated on selective media. Yeast cultures were grown to log phase and harvested when they reached a density of 4.5 OD by spinning at 3,000 × *g* for 10 min. Total RNA was isolated from the cell pellet by standard methods of hot acid phenol RNA isolation and subsequent ethanol precipitation. Smaller RNAs, such as tRNA, were enriched from this total RNA pool using the PureLink miRNA Isolation Kit (Invitrogen) and stored at −80 °C.

### RNA Degradation Assays.

Assays to detect polyadenylation or exoribonuclease activity with unmodified tRNA used 5 nM enzyme with 20 nM unlabeled or 5′-fluorescein–labeled RNA, respectively. For reactions using purified components that were mixed together, equimolar quantities of cofactors (Mpp6, Rrp47, or TRAMP components) were added to reconstituted exosomes Exo11^Dis3/Rrp6^ or Exo13^Dis3/Rrp6/Rrp47/Mpp6^ whenever applicable and then incubated on ice for 30 min before being added to the reaction mix. All reactions were performed in reaction buffer (100 mM NaCl, 20 mM Tris⋅Cl pH 7.5, 0.5 mM DTT, 1 mM MgCl_2_, 1 mM ATP, and 1 U/μL RNase inhibitor [New England BioLabs]) and incubated at 30 °C for the indicated times. At different time points, 12-µL aliquots from the reaction were added to 2 µL of proteinase K (New England BioLabs) (final concentration 0.2 mg/mL), followed by incubation at 37 °C for 30 min to stop the reaction by degrading the protein components, after which an equal volume of loading dye containing formamide, 10 mM EDTA, and 0.01% xylene cyanol was added to the quenched aliquot. The reaction products were electrophoresed using 15% acrylamide/8 M urea gels (Invitrogen) in 1× TBE and scanned using a Typhoon FLA9500 (GE Healthcare) to produce a raw .gel file and a .tif file per scan that were then used for analysis. Products of ribonuclease activity were quantified as described previously (12) by integrating densities from the .tif output format, while figures displaying images used the .gel output format with an enhanced signal-to-noise ratio for better visualization. To quantify Dis3 exoribonuclease activity, a fraction of full-length RNA substrate degraded at a given time was calculated based on the quantity of 4- to 5-nt decay products detected by densitometry using ImageJ. This fraction was converted to nM via a standard curve of 4- to 5-nt decay products that were generated by completely degrading the tRNA. Data from triplicate experiments were analyzed for each sample, and initial rates of Dis3-product formation were calculated from the slope of line-fitting data points obtained within the linear range.

For calculation of half-lives, the rate of substrate decay was calculated by quantifying the amount of substrate remaining using ImageJ, plotting against time, and then fitting data to either a single or double exponential equation using GraphPad Prism 8. For the single exponential decay model, the equation is Y = (Y0 - Plateau)*exp(-K*X) + Plateau, where Y0 is the Y value when X (time) is 0, Plateau is the Y value at infinite times, K is the rate constant, and half-life is computed as ln (2)/K. For the double exponential decay model, which accounts for a fast phase and a slow phase, the equation is Y = Plateau + SpanFast*exp(-KFast*X) + SpanSlow*exp(-KSlow*X). SpanFast = (Y0-Plateau)*PercentFast*0.01 and SpanSlow = (Y0-Plateau)*(100-PercentFast)*0.01, where Y0 is the Y value at time 0, Plateau is the Y value at infinite times, KFast and KSlow are the two rate constants, and t_1/2_ Fast and t_1/2_ Slow are the corresponding half-life values, computed as ln (2)/K. PercentFast, which is accounted for by the faster of the two components, refers to the fraction from Y0 to Plateau. For unmodified tRNA_i_^Met^, substrate and products were detected by monitoring the fluorescence of fluorescein. For native tRNA_i_^Met^ and total yeast RNA (100 ng), gels were stained with SYBR Gold (Invitrogen) following the manufacturer’s protocol and scanned using a Typhoon FLA9500 (GE Healthcare) to produce a raw .gel file and a .tif file per scan, which were then used for analysis of substrate stability and/or decay.

### HPLC Analysis of Dis3 Decay Products.

Decay products were detected by analyzing the products of RNA decay reactions that contained 2 µM enzyme and 10 µM native or unmodified tRNA_i_^Met^ in 100 mM NaCl, 20 mM Tris⋅Cl pH 7.5, 0.5 mM TCEP, and 5 mM ATP·MgCl_2_ after incubation at 30 °C for 1 h. Reactions were stopped by addition of proteinase K to a final concentration of 0.5 mg/mL and incubation at 37 °C for 30 min to degrade proteins in the reaction. Samples were mixed with an equal volume of 2× HPLC running buffer A (50 mM potassium phosphate pH 7.0 and 10 mM tetrabutylammonium hydrogensulfate [Sigma-Aldrich]). Products were then separated by ion-pair reverse-phase HPLC (Nova-Pak C18, 60 Å, 4 mm, 3.9 × 150-mm column) by running in buffer A at a flow rate of 1 mL/min at 40 °C and eluted with 100% buffer B (buffer A containing 50% [vol/vol] acetonitrile) from 5 min to 30 min (12). UV absorbance at 260 nm was monitored to detect nucleotides or RNA products.

### Yeast Strains.

Yeast growth media, including rich (YPD; yeast extract, peptone, dextrose) and minimal (SD) media, were prepared as described previously, and standard protocols were used for the genetic manipulation of *S. cerevisiae* (80). All yeast pRS vectors were generated using standard cloning approaches. The *RRP6* open reading frame (ORF) and all *rrp6* alleles were PCR-amplified from *S. cerevisiae* genomic DNA with oligonucleotides containing EcoRI and SalI restriction sites at the 5′ and 3′ ends, respectively, and then inserted into the pRS413 vector. All *rrp6* PCR constructs contain a stop codon at the 3′ end. A promoter sequence corresponding to the first 129 bp upstream of the endogenous *RRP6* ORF was synthesized as a XbaI/EcoRI geneBlock (IDT) and inserted into each pRS vector containing *RRP6* or *rrp6* alleles. The *MTR4* ORF was generated as a EcoRI/SalI PCR fragment from yeast genomic DNA and inserted into a pRS415 or a pRS416 vector. All *mtr4* alleles contain a stop codon at their 3′ end. A 500-bp fragment corresponding to the promoter sequence immediately upstream of the endogenous *MTR4* ORF was PCR-amplified using XmaI and EcoRI restriction sites, and a 200-bp fragment corresponding to the terminator region immediately downstream of the *MTR4* stop codon was PCR-amplified using SalI and XhoI restriction sites on the 5′ and 3′ oligonucleotides, respectively, and inserted into pRS415 and pRS416 *MTR4* vectors.

The haploid strain containing the *rrp6Δ::KanMX* gene deletion and the MTR4/*mtr4Δ* diploid strain were obtained from the *Saccharomyces* Gene Deletion Project collection (Open Biosystems) and were in the BY4741/BY4743 background (S288C, *his3Δ1 leu2Δ0 ura3Δ0 met15Δ0*). The *rrp6Δ mtr4Δ* double-knockout strain was generated while covered with a pRS416 *MTR4* plasmid because *MTR4* is essential for yeast viability. The MTR4/*mtr4Δ* diploid was transformed with the pRS416 *MTR4* plasmid, then sporulated and dissected to obtain the *mtr4Δ* + pRS416 *MTR4* haploid (MATα type). This strain was crossed to the MATa *rrp6Δ::kanMX* strain. The resulting heterozygous diploid was sporulated to obtain the *rrp6Δ mtr4Δ* + pRS416 *MTR4* double mutant by standard tetrad dissection. Both *rrp6Δ mtr4Δ* deletions were verified by PCR genotyping. This parental strain was used to obtain viable *rrp6Δ mtr4Δ* + pRS416 *MTR4* strains containing various combinations of pRS413 *rrp6* alleles and pRS415 *MTR4* vector. The *rrp6* alleles used in this study were pRS413 *RRP6*, pRS413 *rrp6*^*exo−*^ (D238N), and pRS413 empty vector with the intent of testing the effects of WT RRP6, exoribonuclease-inactive Rrp6, or yeasts lacking Rrp6, respectively. The parental strain, *rrp6Δ mtr4Δ* + pRS416 *MTR4*, was transformed with plasmids and plated on selective SD-Ura-His-Leu solid medium for viable colonies. Attempts to obtain strains with inactivating mutations in both Rrp6 and Mtr4 failed, suggesting that inactivating mutations in Rrp6 cannot suppress the essential functions of Mtr4 in vivo.

Individual clones of strains *rrp6Δ mtr4Δ* + pRS416 *MTR4* + pRS415 *MTR4* containing pRS413 *rrp6* alleles were then selected and grown overnight at 30 °C in selective SD-His-Leu-Ura medium. The next day, cells were diluted to an OD_600_ of 0.2 and grown until they reached midlog phase, and two or three OD of cells were harvested by spinning at 3,000 × *g* for 5 min at 4 °C. Cell pellets were washed in sterile nuclease-free water, spun again to remove supernatant, snap-frozen in liquid N_2_, and stored at −80 °C. Total RNA was isolated from the cell pellets using the MasterPure Yeast RNA Purification Kit (Lucigen), resuspended in nuclease-free water, and stored at −80 °C.

### RNA-Sequencing Sample Preparation and Analysis.

RNA samples extracted from yeast strains were subjected to RiboGreen quantification for quality control as assayed with an Agilent BioAnalyzer 4. Then 1 µg of total RNA with an RNA integrity number varying from 6.1 to 8.9 underwent ribosomal depletion with the Ribo-Zero Gold rRNA Removal Kit (Yeast) (Illumina; catalog no. MRZY1306). Libraries were prepared using the TruSeq Stranded Total RNA LT Kit (Illumina; catalog no. RS-122-1202) according to the manufacturer’s instructions with eight PCR cycles. Samples were barcoded and analyzed using an Illumina HiSeq 4000 in a 50-bp/50-bp paired end run using the Illumina HiSeq 3000/4000 SBS Kit. On average, 61 million paired reads were generated per sample.

The output data (FASTQ files) were mapped to the target genome (sacCer3) using the rnaStar aligner version 2.5.0a (81) that maps reads to the genome. The reads were mapped twice using the two-pass mapping method (82). The first mapping pass used a list of known annotated junctions from Ensemble version 94. Novel junctions found in the first pass were then added to the known junctions, and a second mapping pass was done, using the RemoveNoncanoncial flag. After mapping, the output SAM files were postprocessed using the Picard tools 1.124 to add or replace read groups, which also sorts the file and converts it to the compressed BAM format. The expression count matrix was then computed from the mapped reads using HTSeq version 0.5.3 (https://www.huber.embl.de/users/anders/HTSeq/doc/overview.html) and *S. cerevisiae* genome version R64-1–1.94.gtf. The raw count matrix generated by HTSeq were then processed using the R/Bioconductor package DESeq (https://www.huber.embl.de/users/anders/DESeq/), which was used to both normalize the full dataset and to analyze differential expressions between sample groups. The data were clustered in several ways using the normalized counts of all genes, which gave a total of 10 counts when summed across all samples. To obtain the normalized bedGraph files, scripts were used to compute the coverage files from RNA-seq BAM files normalized to total unique counts (counts/million) and converted to the compressed bedGraph format. Normalized coverage plots were then visualized and compared across different samples using the Integrative Genomics Viewer.

## Supplementary Material

Supplementary File

## Data Availability

RNA sequencing data from this study have been deposited in the Gene Expression Omnibus (GEO) database, https://www.ncbi.nlm.nih.gov/geo (accession no. GSE160368).
