# Supplementary material for: Substrate discrimination and quality control require each catalytic activity of TRAMP and the nuclear RNA exosome
Source: Proc Natl Acad Sci U S A. 2021 Mar 29;118(14):e2024846118. doi: 10.1073/pnas.2024846118 (PMC8040639; doi:10.1073/pnas.2024846118)

PNAS
www.pnas.org

SUPPLEMENTARY INFORMATION

Substrate discrimination and quality control require each catalytic activity of TRAMP and the nuclear RNA exosome

Mom Das[1], Dimitrios Zattas[1], John C. Zinder[1,2], Elizabeth V. Wasmuth[1], Julien Henri[1,3], Christopher D. Lima[1,4*]

[1]Structural Biology Program, Sloan Kettering Institute, Memorial Sloan Kettering Cancer Center, New York, New York 10065, USA

[2] Tri-Institutional Training Program in Chemical Biology, Memorial Sloan Kettering Cancer Center, New York, New York 10065, USA

[3]Present address: Laboratoire de Biologie Moléculaire et Cellulaire des Eucaryotes, Institut de Biologie Physico-Chimique, Unité Mixte de Recherche 8226 CNRS Sorbonne Université, 13 rue Pierre et Marie Curie, 75005 Paris, France

[4]Howard Hughes Medical Institute, 1275 York Avenue, New York, NY 10065, USA

[*]To whom correspondence should be addressed: limac@mskcc.org

Running Title: Quality control and the RNA exosome

Supplementary Figures

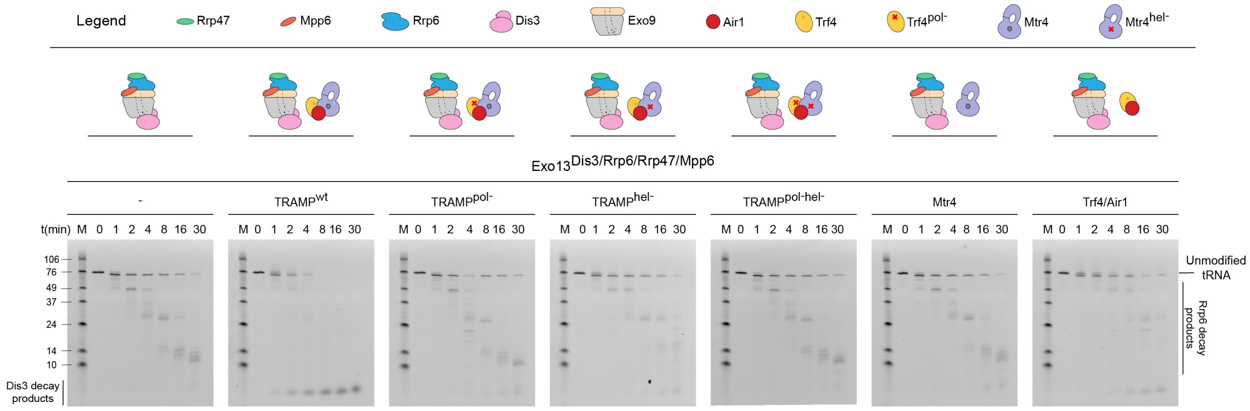

Fig. S1. Degradation of unmodified tRNA by the nuclear exosome and components of TRAMP.

Gel images illustrating a time course for degradation of unmodified 5′-fluor-$tRNA_i^{Met}$ by $Exo13^{Dis3/Rrp6/Rrp47/Mpp6}$ in the absence or presence of TRAMP, TRAMP variants lacking helicase or polymerase activities, or individual components of TRAMP (Mtr4 or Trf4/Air1). RNA degradation products are indicated for Dis3 or Rrp6-mediated decay. Representative gels shown for assays that were performed in triplicate. Legend and cartoons above gel images depict composition of complexes used in each assay. Red crosshair indicates a mutation in the active site as described in the main text.

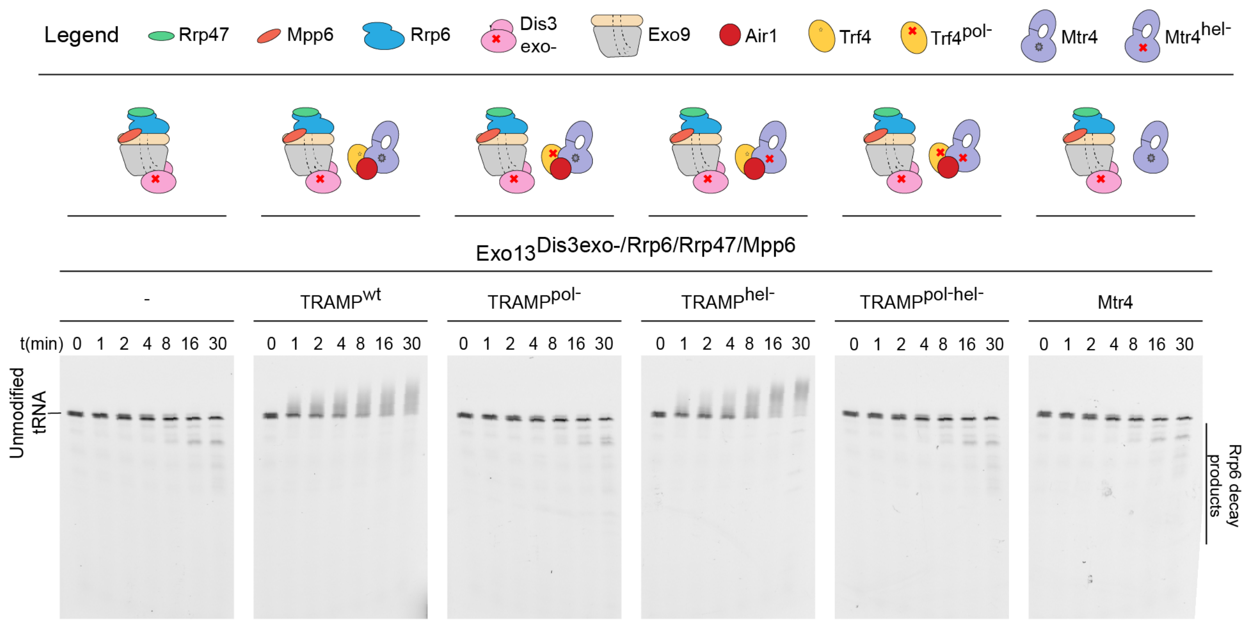

Fig. S2. Dis3 exoribonuclease activity is required for degradation of unmodified tRNA. Gel images illustrating a time course for degradation of unmodified 5′-fluor-tRNA$_i^{Met}$ by Exo13$^{Dis3exo-/Rrp6/Rrp47/Mpp6}$ in the absence or presence of wild-type TRAMP, polymerase or helicase deficient TRAMP, or Mtr4. Representative gels shown for assays that were performed in triplicate. Legend and cartoons above gel images depict composition of complexes used in each assay. Red crosshair indicates a mutation in the active site as described in the main text.

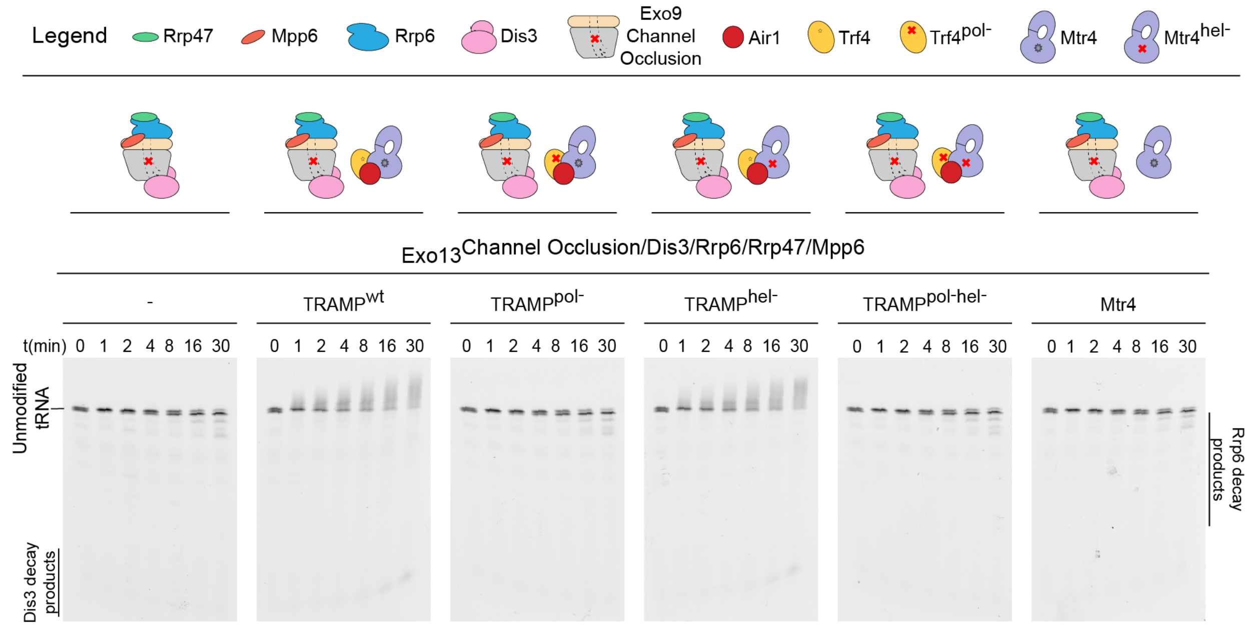

Fig. S3. Channel occlusion disrupts degradation of unmodified tRNA.

Gel images illustrating a time course for degradation of unmodified 5′-fluor-$tRNA_i^{Met}$ by $Exo13^{Channel\ Occlusion/Dis3/Rrp6/Rrp47/Mpp6}$ in the absence or presence of wild-type TRAMP, polymerase or helicase deficient TRAMP, or Mtr4. Representative gels shown for assays that were performed in triplicate. Legend and cartoons above gel images depict composition of complexes used in each assay. Red crosshair indicates a mutation in the active site as described in the main text.

A

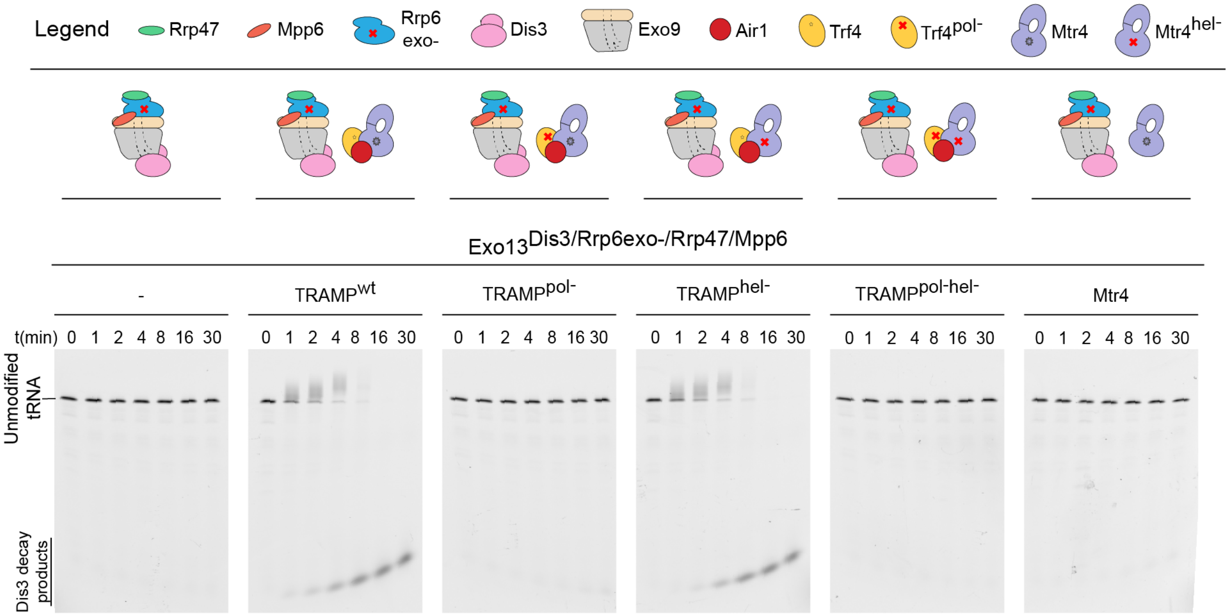

B

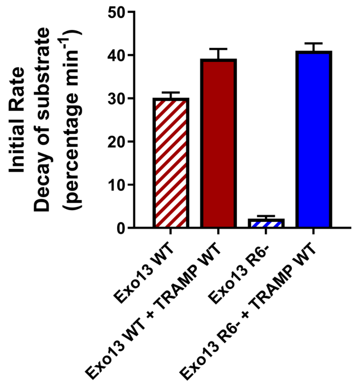

Fig. S4. Rrp6 inactivation enables helicase-independent decay of unmodified tRNA. (*A*) Gel images illustrating a time course for degradation of unmodified 5′-fluor-$tRNA_i^{Met}$ by $Exo13^{Dis3/Rrp6exo-/Rrp47/Mpp6}$ in the absence or presence of wild-type TRAMP, TRAMP variants lacking helicase or polymerase activities, or Mtr4. Representative gels shown for assays that were performed in triplicate. Legend and cartoons above gel images depict composition of complexes used in each assay. Red crosshair indicates a mutation in the active site as described in the main text.

(*B*) Bar graph illustrating initial rates of substrate decay, as calculated from the substrate remaining at each time point in the linear range of the assay, of unmodified 5′-fluor-$tRNA_i^{Met}$ by wild-type Exo13 or $Exo13^{R6exo-}$ that lacked Rrp6 catalytic activity in absence or presence of wild-type TRAMP as obtained from data depicted in panel *A* and from Fig. S1. Dashed bar indicates that products are consistent with Rrp6-mediated degradation, solid bar indicates that products are consistent with Dis3-mediated degradation. Assays performed in triplicate, mean values are shown with error bars at ±1 SD.

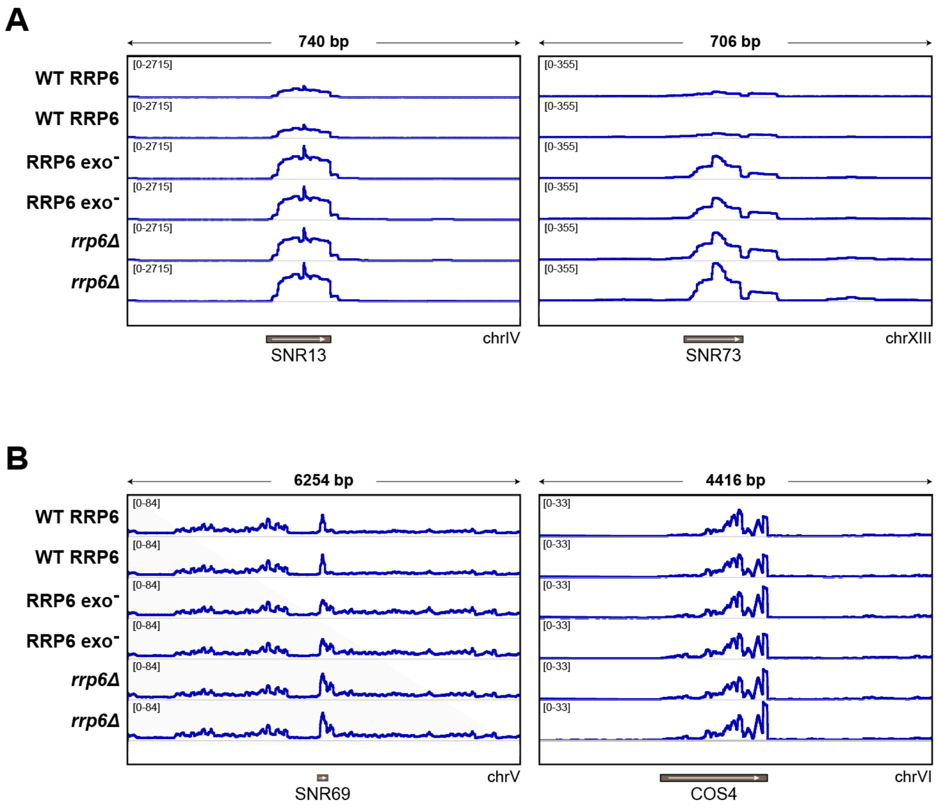

Fig. S5. Comparison of RNA abundance between Rrp6 and Rrp6 variants.

IGV browser tracks of (*A*) *SNR13* and *SNR73*, and (*B*) *SNR69* and *COS4* representing RNA sequencing data derived from yeast strains with wild-type Rrp6 (WT), exoribonuclease inactive (Rrp6 exo-) or Rrp6 knockout (*rrp6Δ*) highlighting (*A*) transcript abundance that is lower only in WT Rrp6 compared to Rrp6 exo- or *rrp6Δ* strains or (*B*) transcript abundance that is similar between the three strains. Data derived from two independent biological replicates. Y-axis represents normalized reads in RPKM (reads per kilobase of transcript per million).

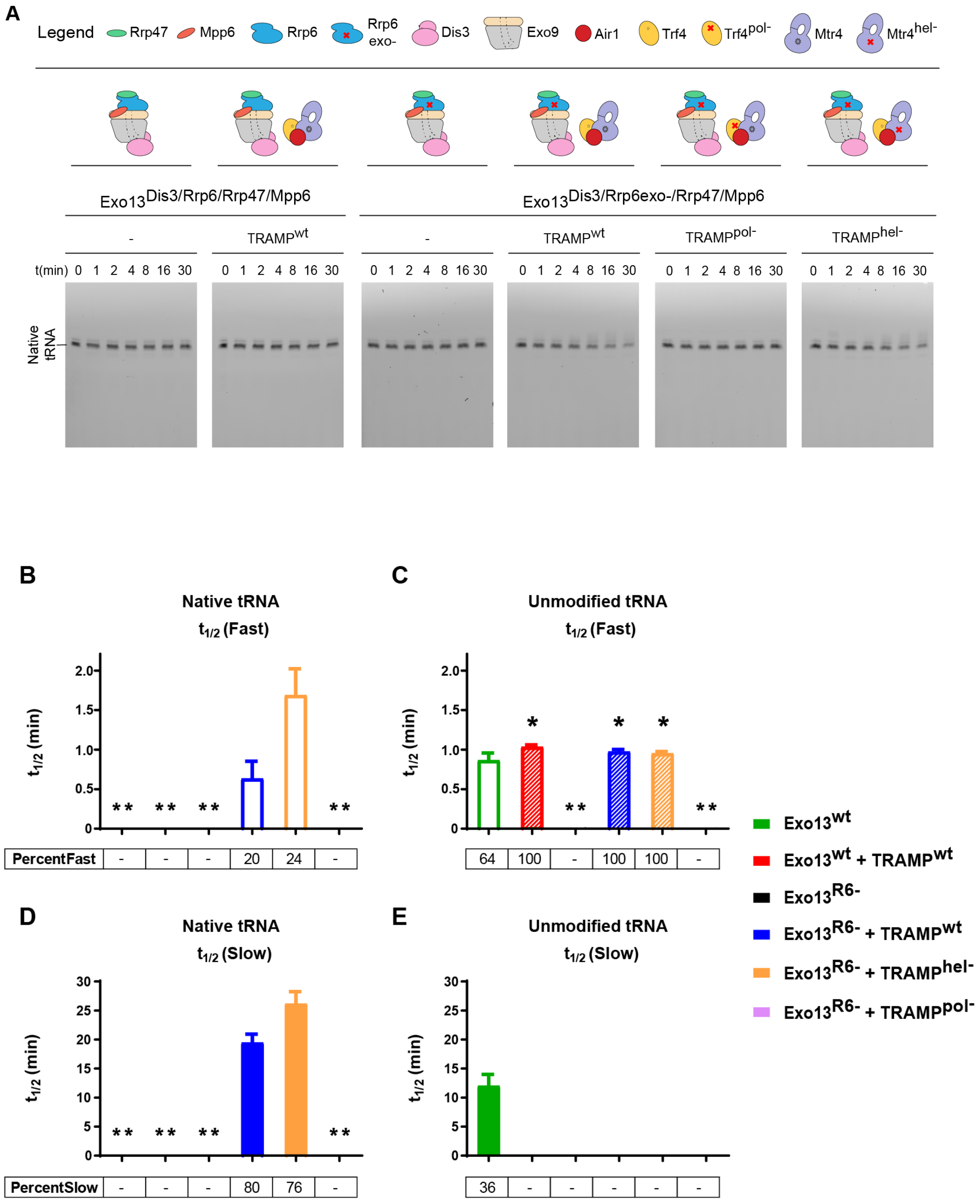

Fig. S6. Degradation of native $tRNA_i^{Met}$ and analysis of half-life.

(*A*) Gel images illustrating a time course for degradation of native $tRNA_i^{Met}$ by $Exo13^{Dis3/Rrp6/Rrp47/Mpp6}$ or $Exo13^{Dis3/Rrp6exo-/Rrp47/Mpp6}$ in the absence or presence of wild-type TRAMP or TRAMP variants lacking helicase or polymerase activities. Representative gels

shown after staining by SYBR-gold for assays that were performed in triplicate. The Dis3-dependent 4-5 nt decay products are too small to be stained with SYBR gold and, hence, are not visible in these gels. Therefore, degradation of the native $tRNA_i^{Met}$ substrate is estimated by monitoring substrate depletion during the time course of the assay and represented with decay plots in Fig. 6*A*. HPLC analysis of Dis3-generated decay products comparing degradation of native and unmodified $tRNA_i^{Met}$ substrates is shown in Fig. 6*D*. Legend and cartoons above gel images depict composition of complexes used in each assay. Red crosshair indicates a mutation in the active site as described in the main text.

(*B*-*E*) Bar graphs indicating the respective half-life ($t_{1/2}$) calculated during degradation of native and unmodified $tRNA_i^{Met}$. Data analyzed by nonlinear regression of results obtained from assays depicted in Figs. 6*A* and 6*B*. A double exponential decay model generates two half-lives from two phases of decay, Fast and Slow. Bar graphs depict the mean values with error bars at ±1 SEM (n=3). An asterisk (*) over bar graphs indicate data analyzed with a single exponential model for unmodified tRNA and indicated TRAMP-RNA exosome complexes; hence represented with only one half-life value in Fig. S6*C*. The percentages of substrate decay corresponding to the phases (fast and slow) are indicated below the bar graphs. A double asterisk (**) replaces bar graphs for reactions with calculated $t_{1/2}$ values for substrates that remained stable over the course of the 30-minute assay. Decay parameters containing mean and standard error of mean (n=3) are reported in Table S1. For further clarity, graph plots illustrating substrate decay under each reaction condition are fitted with both single and double exponential models and represented in Fig. S7.

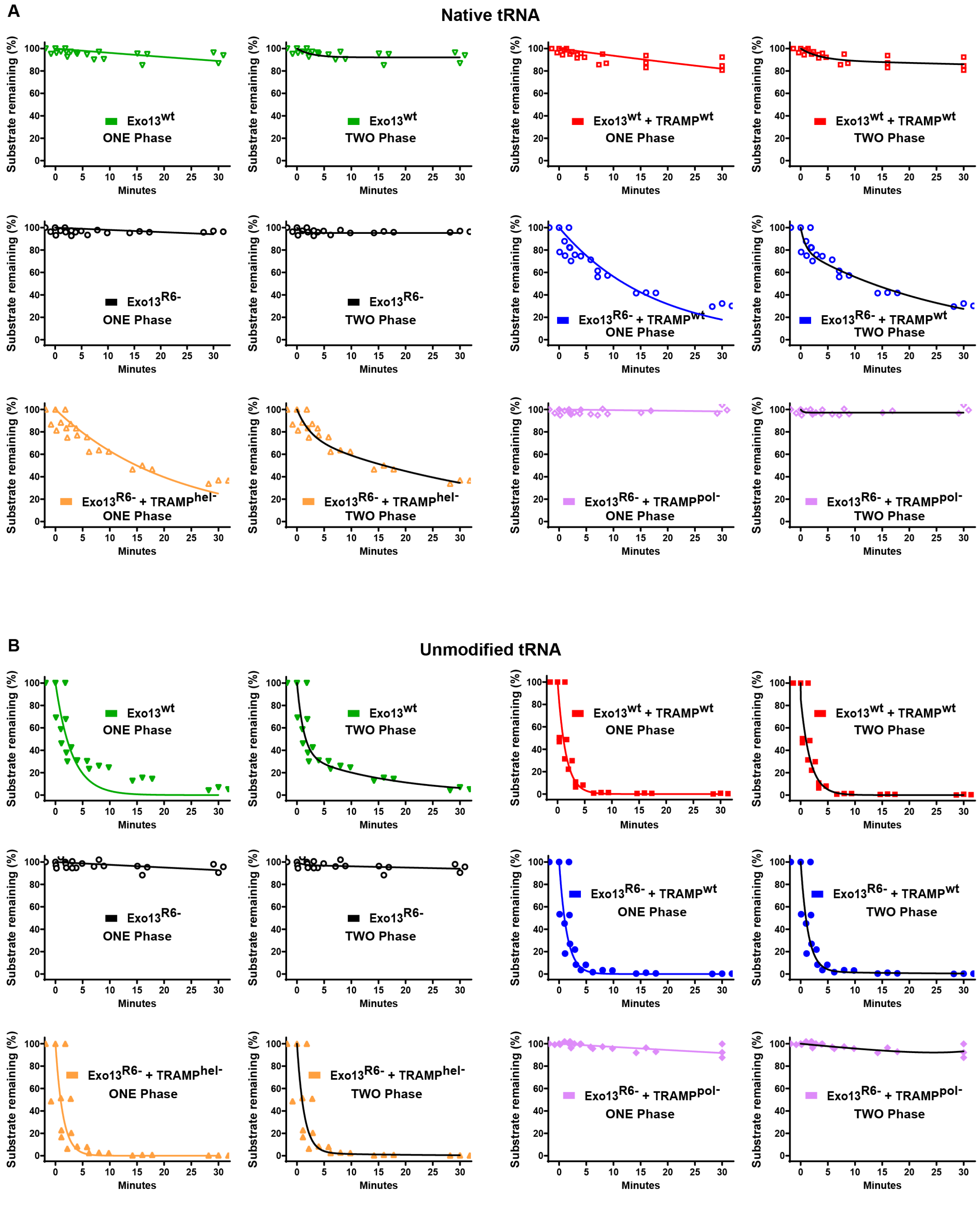

Fig. S7. Analysis of tRNA decay plots.

Graphs depicting substrate remaining from a time-course for degradation of (*A*) native tRNA$_i^{Met}$ or (*B*) unmodified 5′-fluor-tRNA$_i^{Met}$ by the indicated nuclear exosome and TRAMP complexes.

Data analyzed by nonlinear regression of results, by either single or double exponential decay model, are obtained from assays depicted in Figs. 6*A* and 6*B* and are indicated in each graph. Individual data at each time point from three independent experiments are shown in staggered display. Decay parameters for these graphs containing mean and standard error of mean (n=3) are reported in Table S1.

Unmodified tRNA	Parameters - ONE phase decay			Parameters - TWO phase decay					
	Half-life (min)				Half-life (min) (Fast)		Half-life (min) (Slow)		
	Mean	SEM	R-squared	Percent Fast	Mean	SEM	Mean	SEM	R-squared
Exo13^{wt}	2.1×10^{0}	2.2×10^{-1}	0.8908	**64**	**8.6×10^{-1}**	**9.3×10^{-2}**	**1.2×10^{1}**	**2.0×10^{0}**	0.9914
Exo13^{wt} + TRAMP^{wt}	**1.0×10^{0}**	**2.6×10^{-2}**	0.9958	14	3.5×10^{-9}	-	1.2×10^{0}	7.0×10^{-2}	0.9972
Exo13^{R6-} + TRAMP^{wt}	**9.7×10^{-1}**	**2.8×10^{-2}**	0.9947	98	9.3×10^{-1}	5.5×10^{-2}	1.3×10^{1}	2.9×10^{1}	0.9952
Exo13^{R6-} + TRAMP^{hel-}	**9.5×10^{-1}**	**2.4×10^{-2}**	0.9959	97	9.0×10^{-1}	4.7×10^{-2}	1.2×10^{1}	1.9×10^{1}	0.9965

Native tRNA	Parameters - ONE phase decay			Parameters - TWO phase decay					
	Half-life (min)				Half-life (min) (Fast)		Half-life (min) (Slow)		
	Mean	SEM	R-squared	Percent Fast	Mean	SEM	Mean	SEM	R-squared
Exo13^{R6-} + TRAMP^{wt}	1.2×10^{1}	1.1×10^{0}	0.8445	20	6.3×10^{-1}	2.2×10^{-1}	**1.9×10^{1}**	**1.5×10^{0}**	0.9804
Exo13^{R6-} + TRAMP^{hel-}	1.5×10^{1}	1.1×10^{0}	0.8867	24	1.7×10^{0}	3.4×10^{-1}	**2.6×10^{1}**	**2.1×10^{0}**	0.9913

Table S1. Parameters obtained from analysis of tRNA half-life.

Data obtained from nonlinear regression of results obtained from assays depicted in Figs. 6*A* and 6*B* and data depicted in Figs. S6*B*-S6*E* and S7. A double exponential decay model generates two half-lives from two phases of decay, Fast and Slow. Percent Fast reports the percentage of data that fits to the Fast phase of decay. With the exception of one instance (reaction with Exo13^{wt}), data for degradation of unmodified $tRNA_i^{Met}$ fit better to a one phase decay model (see graphs in Fig. S7). Numbers highlighted in bold represent values discussed in the text as these values represent the half-life for the majority of substrate. Numerical values are not reported for substrate half-life when substrates remained stable during the course of the

assay. For unmodified tRNA, those reactions include $Exo13^{R6-}$ and $Exo13^{R6-}$ + $TRAMP^{pol-}$ and for native tRNA substrate, those reactions include $Exo13^{wt}$, $Exo13^{wt}$ + $TRAMP^{wt}$, $Exo13^{R6-}$, and $Exo13^{R6-}$ + $TRAMP^{pol-}$.

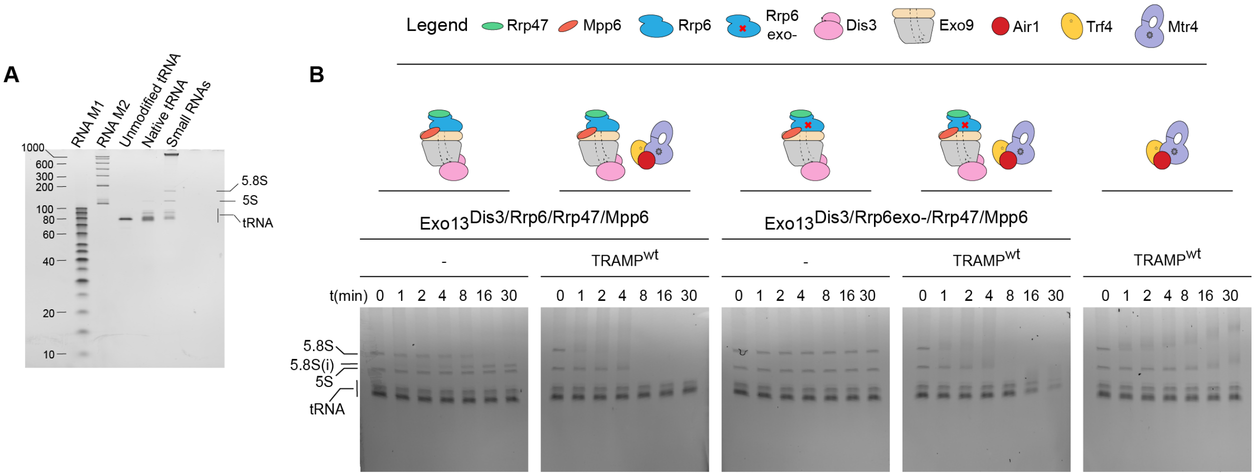

Fig. S8. Degradation of RNA isolated from yeast.

(*A*) Gel depicting extracted RNA with tRNA and other indicated small RNAs after staining with SYBR-gold. RNA markers include RNA M1 (10-100 nt, Alfa Aesar, cat # J76410) and RNA M2 (100-1000 nt, Thermoscientific, cat # SM1833) alongside a sample of unmodified and native $tRNA_i^{Met}$.

(*B*) Gel images illustrating a time course for degradation of RNA by $Exo13^{Dis3/Rrp6/Rrp47/Mpp6}$ or $Exo13^{Dis3/Rrp6exo-/Rrp47/Mpp6}$ in the absence or presence of TRAMP. Gel image on the right shows a reaction containing only TRAMP. Representative gels shown after staining by SYBR-gold for assays that were performed in triplicate. Legend and cartoons above gel images depict composition of complexes used in each assay. Red crosshair indicates a mutation in the active site as described in the main text.

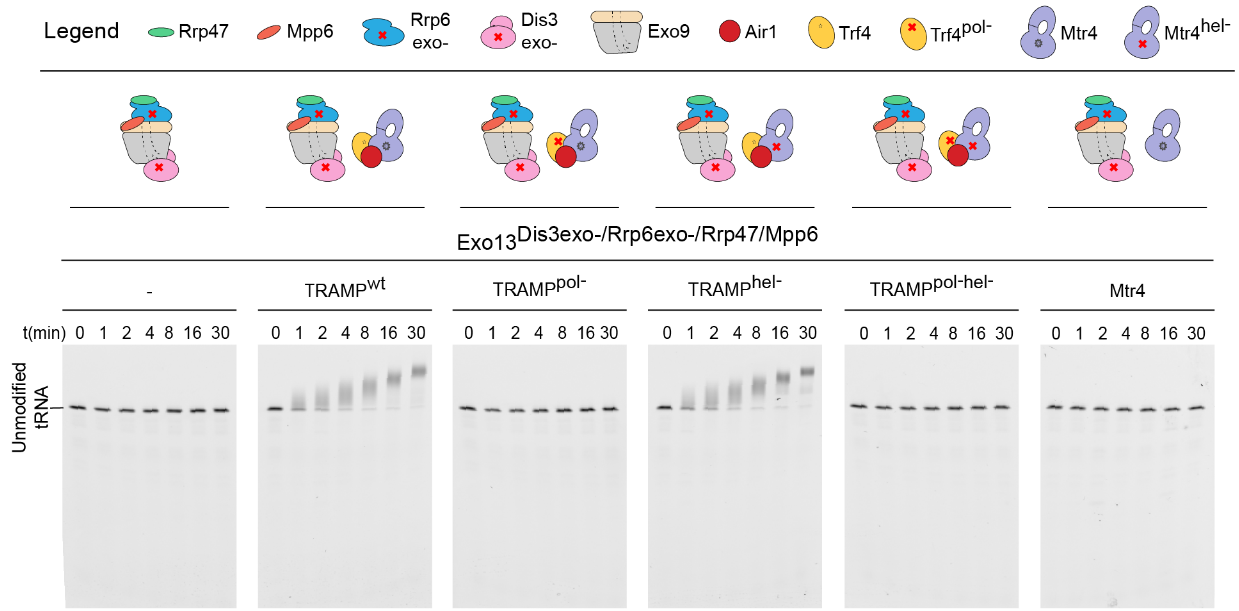

Fig. S9. Exosome catalytic activities are required for degradation of unmodified tRNA. Gel images illustrating a time course for degradation of 5′-fluor-tRNA$_i^{Met}$ by Exo13$^{Dis3exo-/Rrp6exo-/Rrp47/Mpp6}$ in the absence or presence of wild-type TRAMP, TRAMP variants lacking helicase or polymerase activities, or Mtr4. Representative gels shown from assays that were performed twice. Legend and cartoons above gel images depict composition of complexes used in each assay. Red crosshair indicates a mutation in the active site as described in the main text.

Supplementary Information II_Uncropped gel pictures used in the figures

Fig. 1A, top

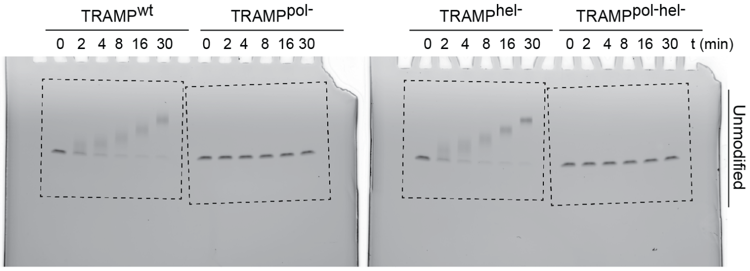

Fig. 1A, bottom

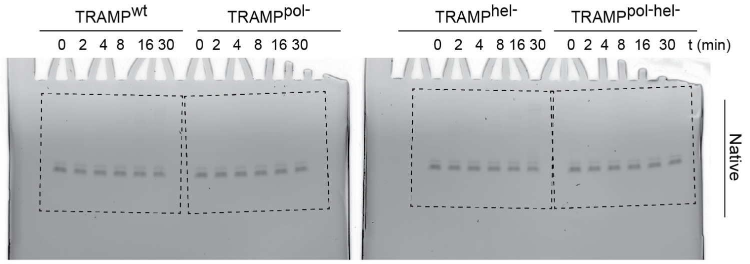

Fig. 2A

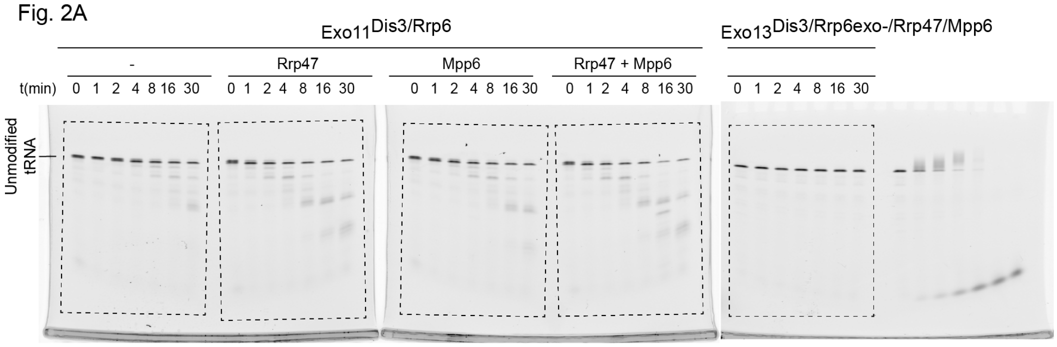

Fig. 2B

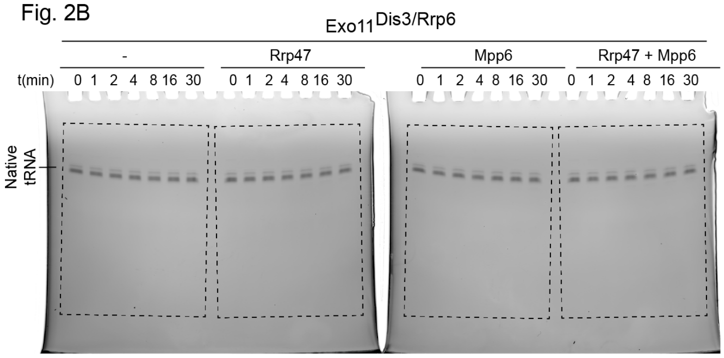

Fig. 3A

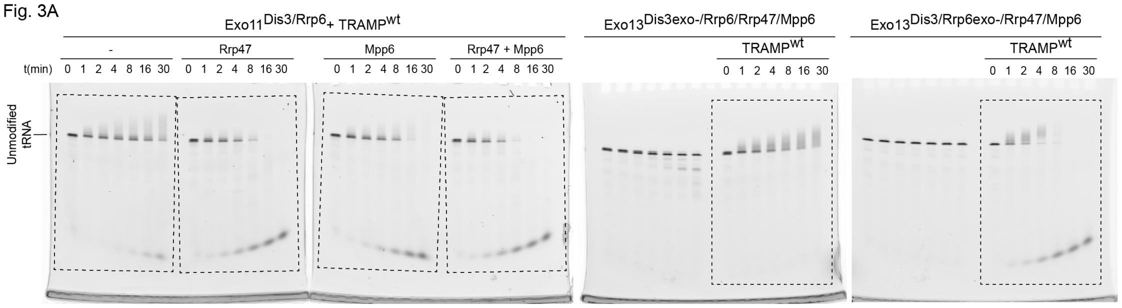

Fig. 3B

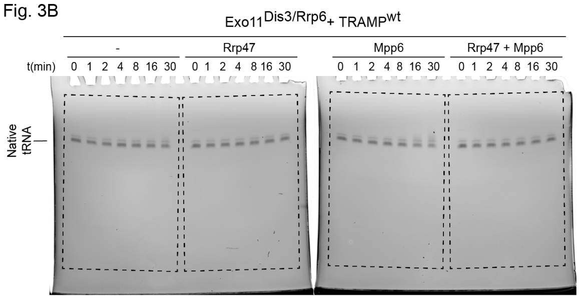

Fig. S1

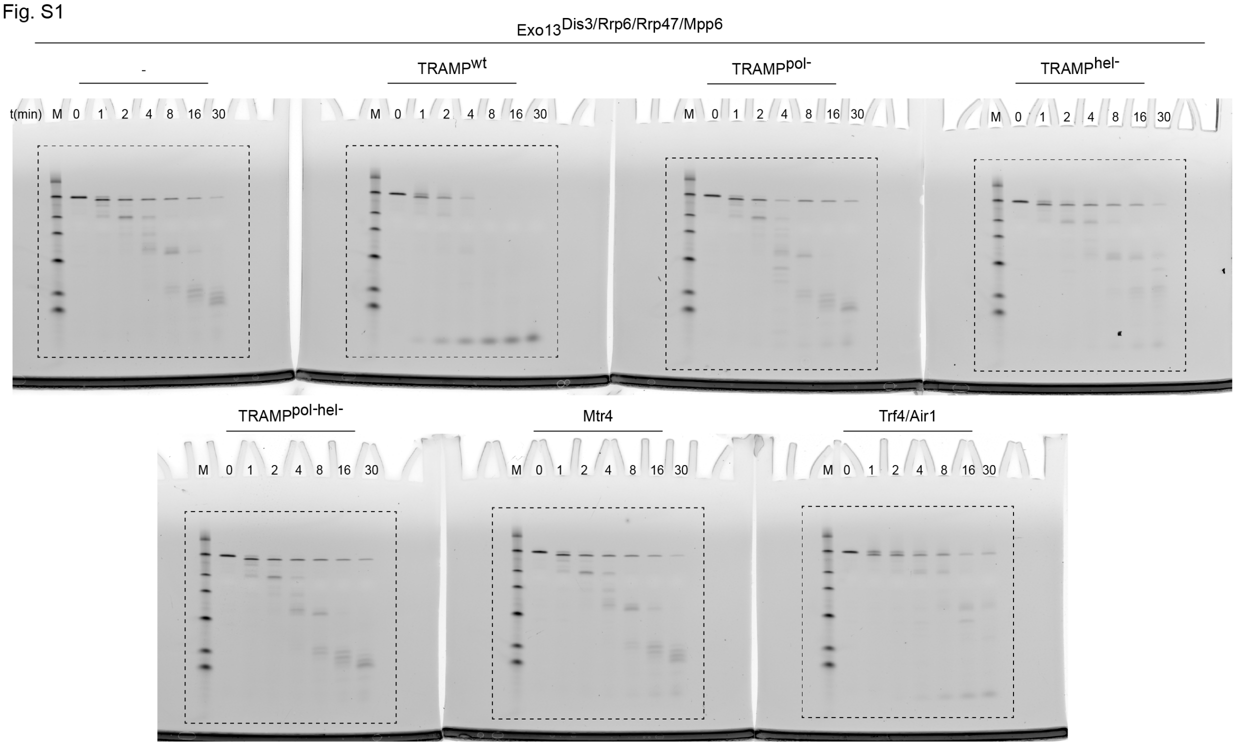

Fig. S2

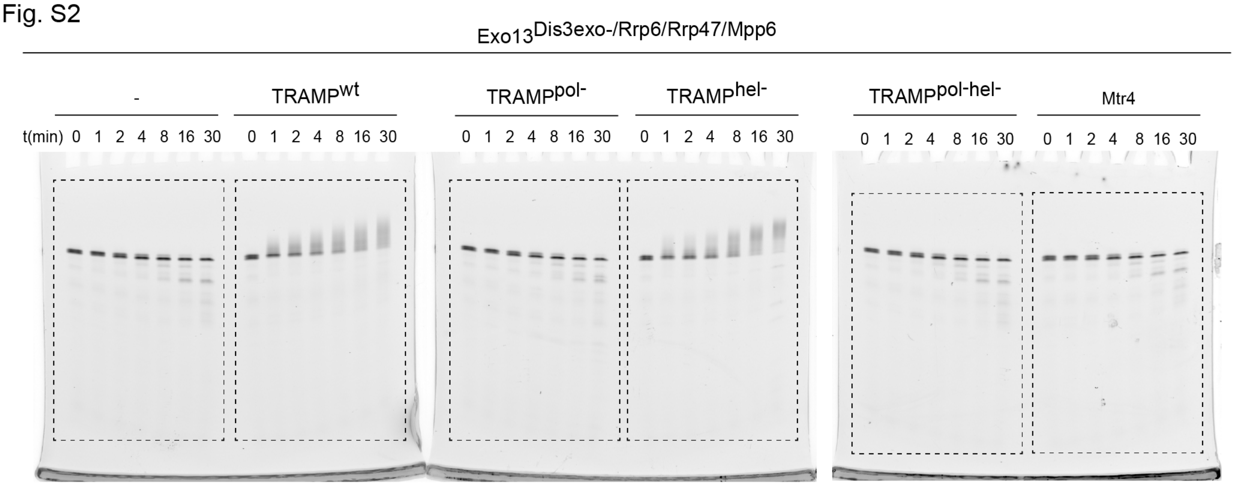

Fig. S3

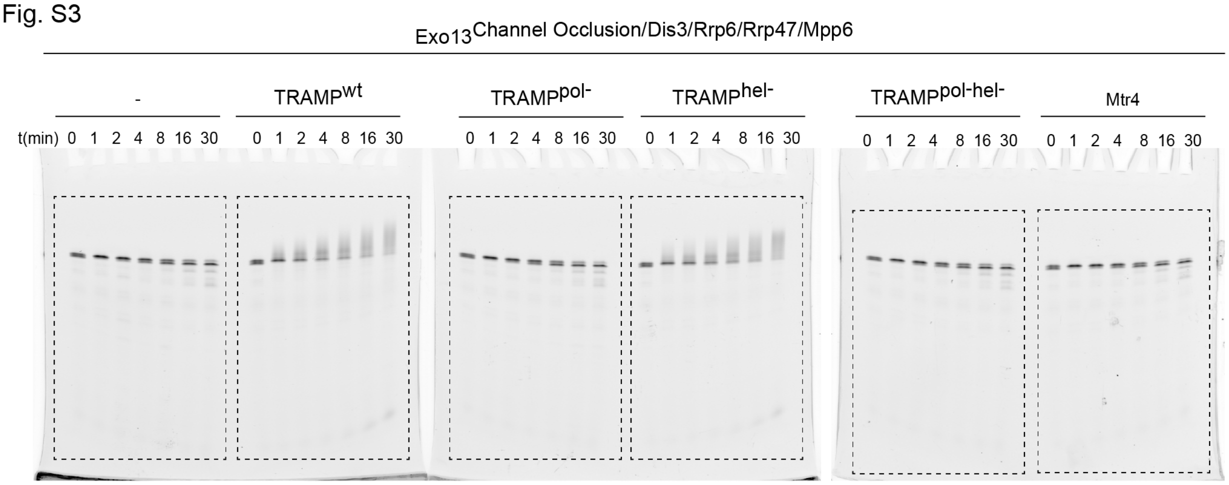

Fig. S4A

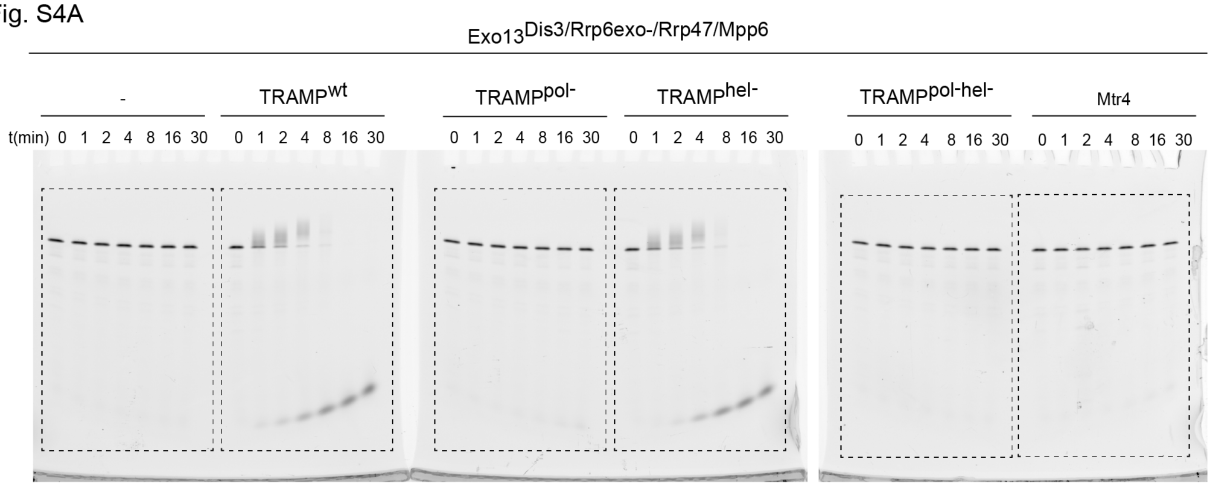

Fig. S6A

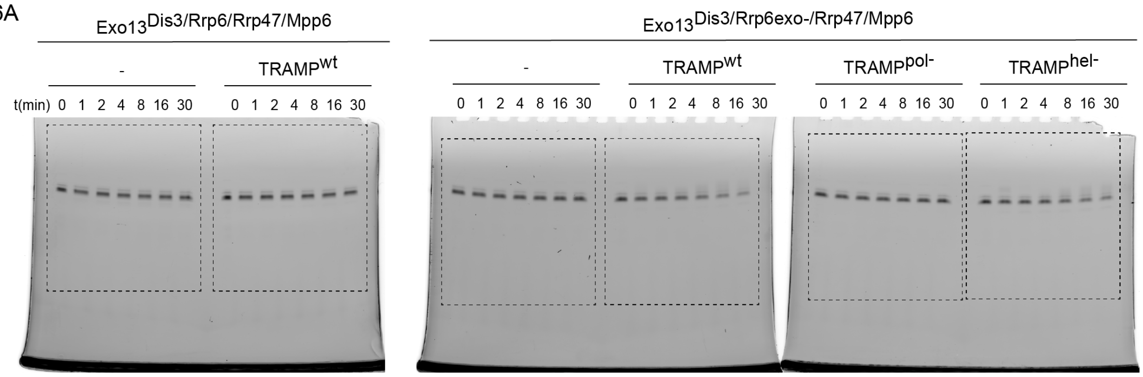

Fig. S8A

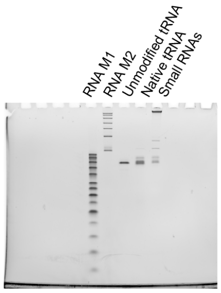

Fig. S8B

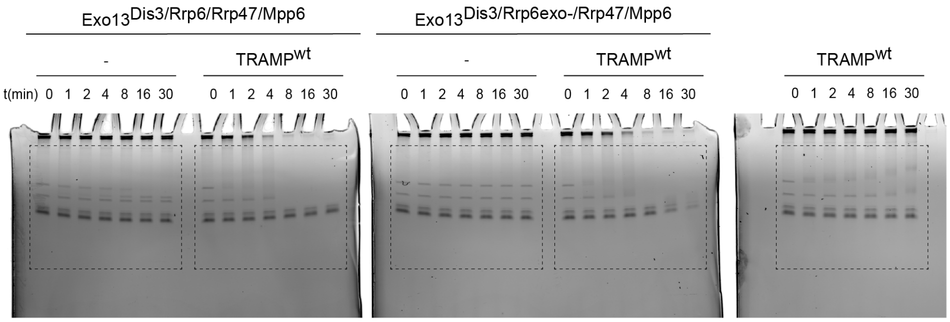

Fig. S9

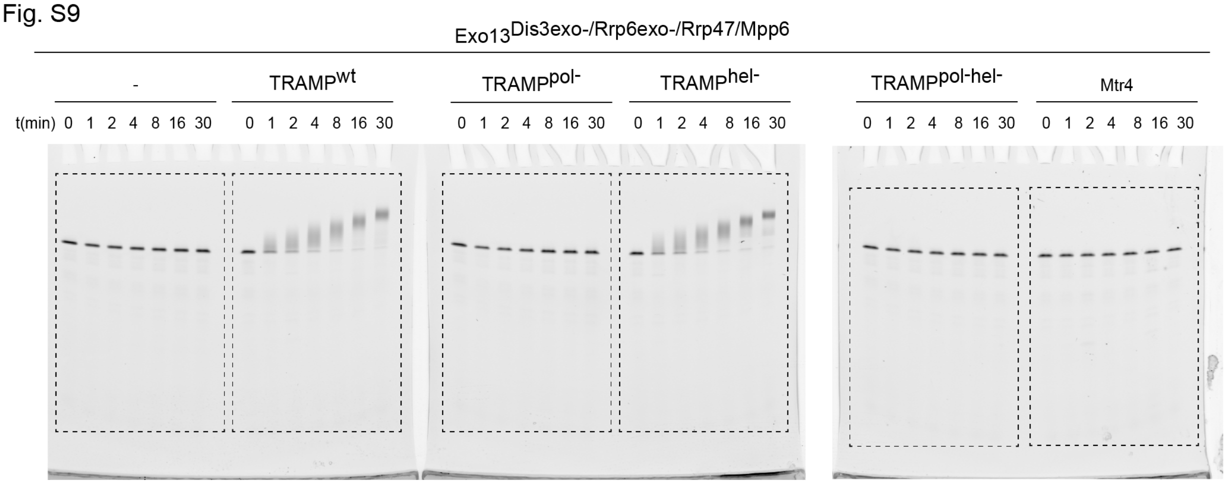

Supplement: Supplementary File [file pnas.2024846118.sapp.pdf]
